# Potential therapeutic targets of the JAK2/STAT3 signaling pathway in triple-negative breast cancer

**DOI:** 10.3389/fonc.2024.1381251

**Published:** 2024-04-18

**Authors:** Lin Long, Xiangyu Fei, Liucui Chen, Liang Yao, Xiaoyong Lei

**Affiliations:** ^1^ School of Pharmaceutical Science, Hengyang Medical School, University of South China, Hengyang, China; ^2^ The First Affiliated Hospital, Hengyang Medical School, University of South China, Hengyang, China; ^3^ Department of Pharmacy, Central Hospital of Hengyang, Hengyang, China

**Keywords:** triple-negative breast cancer, receptor tyrosine kinase, Janus Kinase 2, signal transducer and activator of transcription 3, small molecule compounds

## Abstract

Triple-negative breast cancer (TNBC) poses a significant clinical challenge due to its propensity for metastasis and poor prognosis. TNBC evades the body’s immune system recognition and attack through various mechanisms, including the Janus Kinase 2 (JAK2)/signal transducer and activator of transcription 3 (STAT3) signaling pathway. This pathway, characterized by heightened activity in numerous solid tumors, exhibits pronounced activation in specific TNBC subtypes. Consequently, targeting the JAK2/STAT3 signaling pathway emerges as a promising and precise therapeutic strategy for TNBC. The signal transduction cascade of the JAK2/STAT3 pathway predominantly involves receptor tyrosine kinases, the tyrosine kinase JAK2, and the transcription factor STAT3. Ongoing preclinical studies and clinical research are actively investigating this pathway as a potential therapeutic target for TNBC treatment. This article comprehensively reviews preclinical and clinical investigations into TNBC treatment by targeting the JAK2/STAT3 signaling pathway using small molecule compounds. The review explores the role of the JAK2/STAT3 pathway in TNBC therapeutics, evaluating the benefits and limitations of active inhibitors and proteolysis-targeting chimeras in TNBC treatment. The aim is to facilitate the development of novel small-molecule compounds that target TNBC effectively. Ultimately, this work seeks to contribute to enhancing therapeutic efficacy for patients with TNBC.

## 1 Introduction

Globally, breast cancer stands as the most prevalent malignant tumor (1). Among its subtypes, triple-negative breast cancer (TNBC), characterized by the absence of estrogen receptor (ER), progesterone receptor (PR), and human epidermal growth factor receptor 2 (HER-2) expression, constitutes approximately 10%-15% of all breast cancer cases (2). The treatment of TNBC presents significant challenges, notably its propensity for early metastasis (3) and a comparatively poorer prognosis than other breast cancer subtypes (4). Current clinical strategies for TNBC primarily employ taxanes and anthracycline-based cytotoxic drugs. However, these approaches frequently encounter obstacles in the form of chemotherapy resistance, and the absence of effective small-molecule targeted therapies, constituting primary impediments in TNBC clinical management (5).

Reassessing classic drug targets is crucial for advancing new precision medicine strategies. The JAK2/STAT3 pathway, clinically validated as a therapeutic target for inflammation-related conditions, has shown promise through its inhibitors in treating inflammatory and autoimmune diseases. This success paves the way for novel clinical therapy developments (6, 7). Extensive research has established a strong association between aberrations in the JAK2/STAT3 signaling pathway and key oncogenic processes such as proliferation, invasion, and metastasis in various malignancies, including TNBC. Notably, activation of this pathway has been observed in multiple solid tumors, TNBC included (8–12). Targeted inhibition of the JAK2/STAT3 signaling has demonstrated efficacy in curtailing TNBC cell proliferation, invasion, and migration (13), knockdown of JAK2 or STAT3 in triple-negative breast cancer cells significantly reduced cell proliferation, invasion and migration (14–21), tumor volume and distant metastasis were significantly inhibited in a mouse model of triple-negative breast cancer with conditional knockout of JAK2 or STAT3 (22–25). Moreover, the downregulation of this pathway has been shown to counteract paclitaxel (PTX) resistance (26). Thus, targeting JAK2/STAT3 emerges as a promising therapeutic strategy for treating TNBC and overcoming challenges associated with PTX resistance.

**Figure 1:**
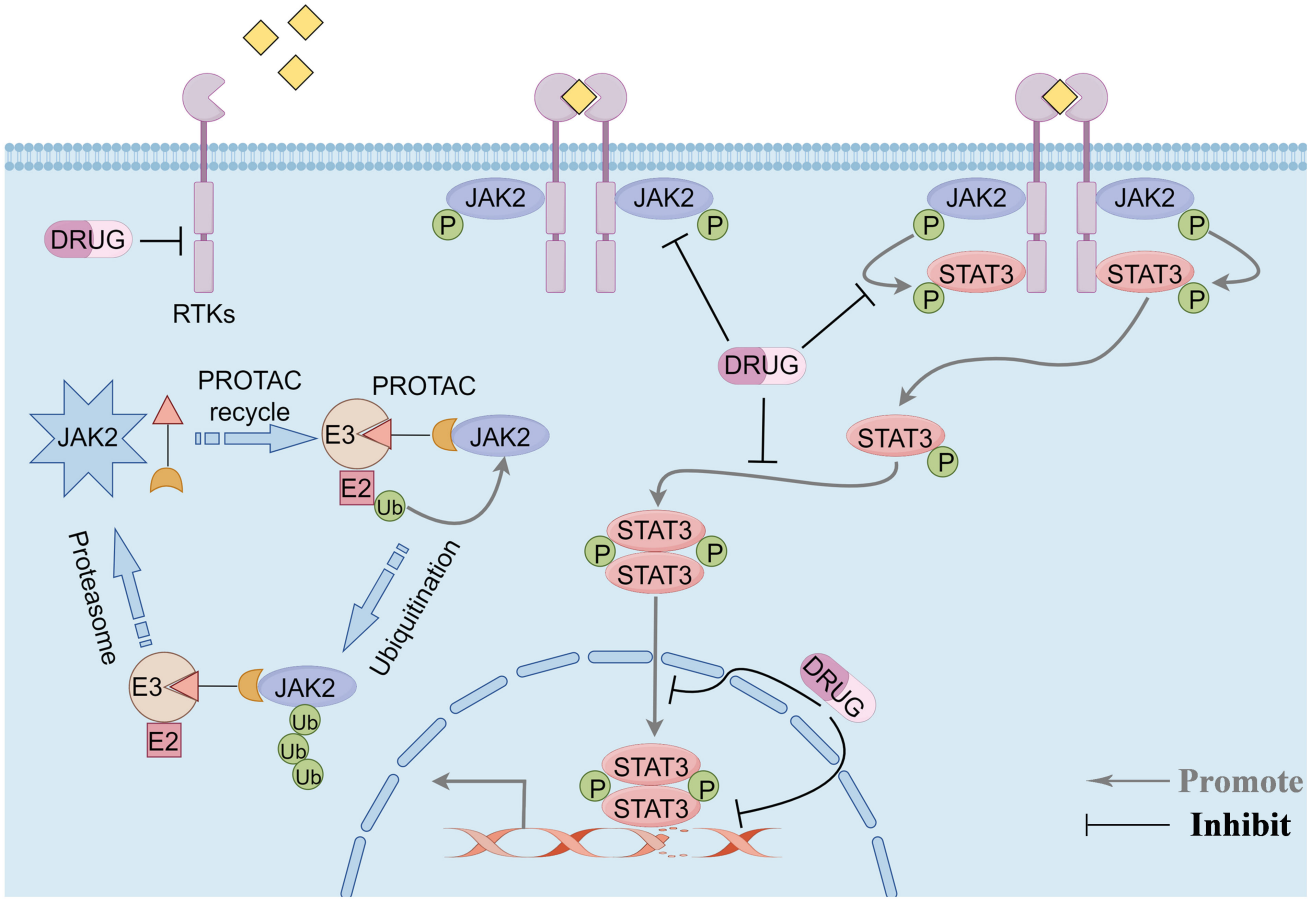
Overview of the JAK2/STAT3 pathway signaling modality and potential therapeutic targets of the pathway. Phosphorylation signaling blockade and protein-targeted degradation pathways. By Figdraw.

The signaling cascade of the JAK2/STAT3 pathway is predominantly mediated through receptor tyrosine kinases (RTKs), JAK2, and the transcription factor STAT3. RTKs are single-pass transmembrane proteins ubiquitously expressed across various cell types, including those within the tumor microenvironment. Characteristically, all RTKs possess a conserved structural composition: an extracellular ligand-binding domain, a transmembrane domain, and an intracellular tyrosine kinase domain (27). Upon ligand binding, RTKs undergo dimerization on the cell membrane and phosphorylate tyrosine residues on the receptors, facilitating their recognition and binding by downstream proteins with SH2 domains, such as JAK2. RTK dimerization brings the associated JAK2 kinase into proximity, enabling their activation through reciprocal tyrosine phosphorylation. Activated JAK2 then stimulates the RTKs to generate binding sites for STAT3. STAT3 binds to RTKs through its SH2 domain and undergoes phosphorylation under the influence of JAK2. The phosphorylated STAT3 forms homodimers and enters the nucleus to induce downstream signal transduction, effectuating various physiological or pathological roles (28). Given the characteristics of this signaling pathway, targeting its components to treat TNBC represents an effective strategy for precision therapy (**Figure 1**).

Consequently, this review systematically summarizes the roles of the JAK/STAT3 pathway in the pathogenesis of TNBC and the current advances in research on small-molecule compounds targeting the JAK/STAT3 signaling pathway as a therapeutic approach for TNBC.

## 2 Receptor tyrosine kinases in TNBC

Receptor tyrosine kinases represent a diverse class of enzyme-linked cell surface receptors with a high affinity for growth factors, cytokines, and hormones. These receptors not only bind specific ligands but also function as protein kinases, phosphorylating tyrosine residues on target proteins. RTKs are categorized into 20 distinct families based on the types of ligands they bind (29). TNBC expresses various RTKs, including epidermal growth factor receptors (EGFRs) (30), vascular endothelial growth factor receptors (VEGFRs) (28), insulin-like growth factor receptors (IGFRs) (31), platelet-derived growth factor receptors (PDGFRs) (32), fibroblast growth factor receptors (FGFRs) (33), leukemia inhibitory factor receptor (LIFR) (34), interleukin-6 cell factor receptor (IL-6R) (35), interleukin-13 cell factor receptor (IL-13R) (36), and glycoprotein 130 receptor (GP130) (37). Before ligand binding, RTKs exist on the cell surface as inactive monomers. Homologous ligand binding induces receptor dimerization, activating their intrinsic kinase activity (38).

### 2.1 Epidermal growth factor receptors: regulator of progression, metastasis, and cancer stem cells in TNBC

Epidermal growth factor receptors, the receptor for epidermal growth factor (EGF), is a key member of the HER family, which also includes Her-2, Her-3, and Her-4 (39). EGF binding to EGFR induces receptor dimerization, a critical step leading to the autophosphorylation of tyrosine residues on the activated receptor. This activation allows the receptor to recruit various signal sequence proteins, transmitting biological signals from the extracellular milieu to the intracellular domain. These signaling cascades culminate in gene transcription, modulating key cellular processes such as proliferation, differentiation, and apoptosis. In cancer, EGFR contributes to tumor progression by promoting invasion and metastasis and stimulating tumor angiogenesis (40). EGFR activates complex signal transduction pathways with primary pathways including mitogen-activated protein kinase (MEK)/extracellular signal-regulated kinase (ERK) (41), JAK2/STAT3 (42), and phosphoinositide 3-kinase (PI3K)/protein kinase B (AKT) (43). Dysregulation of these pathways is intricately linked to tumor development, invasion, and metastasis. EGFR is overexpressed in several malignant tumors, including lung, colon, liver, and breast cancers (44–47). In the context of cancer prognosis, EGFR overexpression is associated with shorter recurrence times, increased recurrence rates, and reduced survival durations (48). In TNBC, the positive expression rate of EGFR is notably higher than in non-TNBC, with over 40% of patients with TNBC exhibiting EGFR overexpression, a factor closely correlated with TNBC prognosis (49, 50). Targeted inhibition of EGFR expression has demonstrated anti-cancer effects in TNBC (51). EGFR expression is also implicated in CD44+ cell aggregation, with its inhibition disrupting cancer stem cell assembly in TNBC. These lines of evidence suggest a link between EGFR expression and the progression of CD44+-mediated cancer stem cells (52).

### 2.2 Vascular endothelial growth factor receptors: regulator of angiogenesis and cancer stem cells in TNBC

Vascular endothelial growth factor receptors, the receptor for vascular endothelial growth factor (VEGF), comprises three primary types: VEGFR1, VEGFR2, and VEGFR3. VEGF induces angiogenesis by binding to VEGFR-2, enhancing the survival, proliferation, migration, and adhesion of endothelial cells (53). However, in pathological contexts, particularly in cancer, VEGFR expression is linked to the promotion of tumor angiogenesis and metastasis (54, 55). Numerous studies have documented the overexpression of VEGFR in a range of malignant tumors, including lung, colon, breast, liver, and ovarian cancers (56–60). In TNBC, elevated VEGF levels correlate with increased metastasis, poor treatment response, and decreased survival rates (61). Upregulation of VEGFR in TNBC is linked to heightened cell proliferation, while its downregulation inhibits this proliferation (62). A notable randomized cohort study has indicated a strong association between high VEGFR expression and 5-year and 10-year breast cancer-specific survival rates in patients (63). These findings underscore the potential of targeting the VEGF/VEGFR axis as a promising approach in the targeted therapy of TNBC. Research employing primary breast cancer mouse models and models of spontaneous breast cancer metastasis has revealed elevated VEGFR expression levels in metastatic breast cancer compared to non-metastatic forms (64). Additionally, VEGFR expression correlates with cancer stem cell characteristics. By activating the VEGFR2/STAT3 pathway, VEGF induces the upregulation of Myc and Sox2 expression, thereby promoting the self-renewal of breast cancer stem cells. The autocrine action of VEGF can establish a positive feedback loop, diminishing the efficacy of anti-angiogenic drugs and enhancing cancer stem cell renewal (28). Consequently, targeting VEGFR expression emerges as a potentially effective therapeutic strategy for the regulation of breast cancer stem cells.

### 2.3 Platelet-derived growth factor receptors: regulator of endothelial cell differentiation and cancer stem cells in TNBC

Tumor blood vessel development is crucial to tumor growth, making angiogenesis a potential target in cancer therapy (65). Platelet-derived growth factor receptors, which binds to platelet-derived growth factor (PDGF), exists in two forms: PDGFRα and PDGFRβ. PDGFR activation, contingent upon PDGF interaction, initiates various intracellular signaling pathways. While PDGFR contributes to vascular repair after tissue damage (66), it also promotes cell proliferation within tumor tissues (67). Studies involving mouse models with differential PDGF gene expression have yielded insightful observations. Specifically, tumors in mice with PDGF gene deficiency exhibit reduced pericyte recruitment, whereas tumors in mice with PDGF overexpression demonstrate increased pericyte recruitment. These findings suggest that tumors recruit pericytes through paracrine PDGF secretion, interacting with PDGFR, facilitating blood vessel maturation, and synergizing with VEGF-mediated angiogenesis, contributing to tumor vascularization (68). Extensive research indicates that PDGFR is overexpressed in various malignant tumors, including lung, colon, breast, and ovarian (69–72). In TNBC, PDGFRβ plays a notable role in mediating endothelial cell differentiation and vasculogenic mimicry in tumor cells (32). Further studies have identified a link between PDGFRβ expression in TNBC, and cancer stem cells, where FOXC2 induces cancer stem cell characteristics and metastasis by upregulating PDGFRβ expression (73). These findings position PDGFR as a promising therapeutic target for TNBC.

### 2.4 Fibroblast growth factor receptors: regulator of cell proliferation and cancer stem cells in TNBC

Fibroblast growth factor receptors, the receptor for fibroblast growth factor (FGF), comprises four subtypes: FGFR1, FGFR2, FGFR3, and FGFR4, collectively forming the FGFR family. Upon binding with FGF, FGFR is activated and modulates multiple intracellular signaling pathways crucial to various biological processes, including angiogenesis and lymphangiogenesis (74). Studies have highlighted the association of FGFR expression in various solid tumors with tumor cell proliferation (75–79). High-throughput sequencing has identified FGFR gene mutations in approximately 7.1% of malignant tumors, with breast cancer exhibiting the second-highest frequency after urothelial carcinoma (80). In TNBC, FGFR3 expression is observed, and inhibition of the FGFR3 signaling pathway reduces TNBC cell invasion and migration (81). Targeting and blocking the FGFR pathway can significantly enhance T cell infiltration and suppress tumor growth in TNBC (33). Some studies have revealed that estrogen can stimulate breast cancer stem cell proliferation via the paracrine FGF/FGFR/T-box transcription factor 3 (TBx3) signaling pathway, and inhibiting this pathway curtails cancer stem cell expansion in TNBC (82). These findings highlight the potential of targeting the FGFR pathway as a therapeutic approach in TNBC.

## 3 Activation of the JAK2/STAT3 signaling pathway in TNBC

Janus Kinase 2, a member of the JAK family of non-receptor tyrosine kinases, includes JAK1, JAK3, and tyrosine kinase 2 (TYK2). JAK3 is predominantly expressed in hematopoietic cells, while JAK1, JAK2, and TYK2 exhibit broader expression across various tissues (83). JAKs mediate a range of disease processes, including immune system disorders (84), hematologic conditions (85), and various malignancies (86, 87). Signal transducer and activator of transcription (STAT) protein family comprises members such as STAT1, STAT2, STAT3, STAT4, STAT5A, STAT5B, and STAT6. Notably, STAT3 is implicated in the promotion of tumor growth and the induction of immunosuppression (88–90). The JAK2/STAT3 signaling pathway is ubiquitously expressed in cells and vital in physiological functions (91), such as cell proliferation, differentiation, apoptosis, and immune regulation (92). Beyond its physiological roles, this pathway is significantly implicated in various pathologies, notably in cancer and autoimmune disorders. In breast cancer, particularly TNBC, JAK2/STAT3 signaling is known for its excessive activation (93). Advanced research has facilitated a more precise molecular classification of TNBC, identifying the mesenchymal subtype characterized by heightened JAK2/STAT3 activity (8). This insight offers a new direction and foundation for the personalized clinical treatment of TNBC, focusing on targeting the JAK2/STAT3 signaling pathway. Research has elucidated that the JAK2/STAT3/Cyclin D2 signaling pathway is pivotal in promoting cancer stem cell proliferation (94). Specifically, in TNBC, studies have demonstrated that downregulating the JAK2/STAT3 pathway can significantly inhibit cancer stem cell proliferation (95). Furthermore, additional research has indicated that suppressing this pathway may reduce TNBC cell proliferation and migration (13).

## 4 The role of the JAK2/STAT3 signaling pathway in multidrug resistance in TNBC

The lack of effective targeted therapies, necessitating reliance on taxanes and anthracycline cytotoxic drugs significantly hinders the treatment of TNBC. However, the emergence of multidrug resistance during treatment poses a formidable challenge to this approach (5). For instance, the activation of the JAK2/STAT3 signaling pathway in nasopharyngeal carcinoma has been demonstrated to induce forkhead box M1 transcription, thereby enhancing resistance to PTX (96). Subsequent studies have revealed the contribution of the JAK2/STAT3 pathway to the development of PTX resistance by upregulating anti-apoptotic gene expression. Targeting this pathway has proven effective in reversing PTX resistance in ovarian cancer (97). In a model of PTX-resistant cells, researchers observed differential expression of the JAK2 gene, suggesting its potential role as a candidate gene linked to PTX resistance in ovarian cancer cell lines (98). Further studies indicate that the downregulation of JAK2/STAT3 signaling pathway can counteract PTX resistance in TNBC (26). Furthermore, a separate research effort found that JAK2 inhibitors can directly bind to the drug efflux protein P-gp in resistant cell lines, thus impeding P-gp-mediated drug efflux (99). Collectively, these studies underscore the significance of the JAK2/STAT3 signaling pathway in the development of multidrug resistance. Consequently, targeting this pathway, either as a standalone therapy or in combination with PTX, presents a promising strategy for the treatment of PTX-resistant TNBC.

## 5 Current therapeutic applications of the JAK2/STAT3 signaling pathway in TNBC

**Figure 2:**
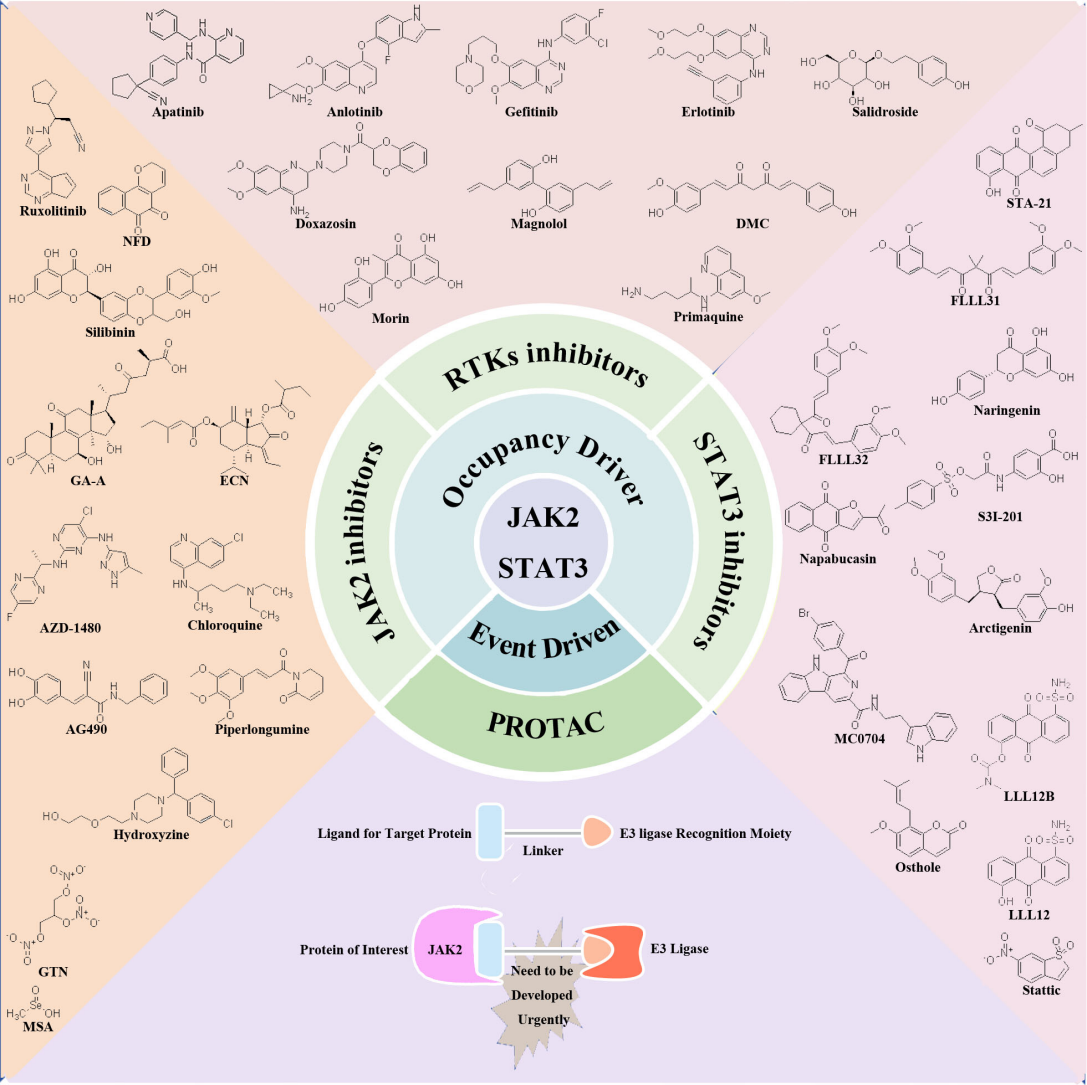
Potential therapeutic targets and inhibitors targeting JAK2/STAT3 signaling pathway for TNBC treatment. Occupation-driven mode: RTKs, JAK2 and STAT3-targeted inhibitors; event driven mode: small molecule compounds targeting JAK2 protein for ubiquitination degradation.

Recent research has elucidated the pivotal role of the JAK2/STAT3 signaling pathway in driving the proliferation, invasion, and migration of TNBC. These findings position the JAK2/STAT3 pathway as a promising therapeutic target for TNBC management. In response to these insights, numerous preclinical and clinical studies are actively exploring the development of inhibitors targeting RTKs, JAK2, and STAT3. These inhibitors are categorized based on their mode of action into traditional small molecule inhibitors in the occupation-driven mode and proteolysis targeting chimera (PROTAC) molecules based on ubiquitin-mediated protein degradation in an event-driven mode. Traditional small molecule inhibitors in the occupation-driven mode function by occupying the active site or binding site of the target protein with small molecule compounds. This action blocks its interaction with downstream signaling molecules, inhibiting its function. On the other hand, PROTAC molecules employ a ligand linker to bind the target protein with an E3 ubiquitin ligase, leveraging the ubiquitin-proteasome system to drive the degradation of the target protein (**Figure 2**).

### 5.1 Occupation driven mode: application of small molecule inhibitors in TNBC

#### 5.1.1 RTKs inhibitors

Currently, the U.S. Food and Drug Administration (FDA) has not approved RTK inhibitors for the treatment of TNBC. Both monoclonal antibodies and small molecule inhibitors are progressing through preclinical and clinical research stages. Preclinical studies have shown that cetuximab can effectively reduce cancer stem cells in TNBC and inhibit tumor growth (100). However, clinical trials reveal a more complex picture. For instance, a study investigating the combination of cetuximab and cisplatin in metastatic TNBC reported benefits in fewer than 20% of patients. Genomic analyses revealed limited efficacy due to cetuximab-induced activation of alternative bypass pathways. Combining cetuximab with inhibitors targeting downstream elements of the EGFR pathway is proposed for enhanced benefits in patients with TNBC (101). Preclinical research has demonstrated that bevacizumab, a VEGFR inhibitor, effectively suppresses TNBC growth *in vivo (* 102). However, the adjunctive use of bevacizumab with chemotherapy did not improve overall survival rates in early-stage patients with TNBC compared to chemotherapy alone. Similarly, tocilizumab, an interleukin-6 receptor (IL-6R) inhibitor, exhibits potential anti-TNBC properties in preclinical studies (103), but its clinical efficacy in TNBC treatment remains unreported and warrants further investigation.

Several RTK small molecule inhibitors have been reported in clinical studies for the treatment of TNBC (**Table 1**). Apatinib, a highly selective VEGFR inhibitor, has exhibited promising efficacy in a Phase II clinical trial for patients with TNBC combined with chemotherapy. The results highlighted not only its effectiveness but also a manageable safety profile (104). Furthermore, combining Apatinib with a programmed death-ligand 1 (PD-L1) inhibitor in another Phase II trial resulted in favorable outcomes with a controllable safety profile (105). Integration of Apatinib with a PD-L1 inhibitor and Eribulin in a multicenter Phase II trial demonstrated significant therapeutic benefits in treating advanced TNBC, notably extending its efficacy to PD-L1–negative patients (106). Anlotinib, identified as a small molecule inhibitor targeting the VEGFR, displayed promising results in advanced TNBC treatment. Specifically, a Phase Ib clinical trial revealed that Anlotinib, when employed in a chemotherapy-free regimen alongside a PD-L1 inhibitor, effectively treated previously advanced patients with TNBC. This combination not only demonstrated favorable efficacy but also maintained a manageable safety profile (107). Another Phase II clinical trial combining Anlotinib with standard chemotherapy for metastatic TNBC demonstrated therapeutic benefits with manageable safety (108).

**Table 1:** Targeting JAK2/STAT3 signaling pathway for TNBC in preclinical studies.

Compd.	Target	Effects/Adverse Reactions	Citation
Apatinib	VEGFR	Combined with chemotherapy in a phase II clinical trial study, excellent results were achieved in patients with TNBC and were safe and manageable; Combination with a PD-L1 inhibitor in a phase II clinical trial study showed favorable results with a manageable safety profile in patients with advanced TNBC; Combination of PD-L1 inhibitors and eribulin shows promising results in the treatment of advanced TNBC in a multicenter phase II clinical trial study.	(104–106)
Anlotinib	VEGFR	Combination with a PD-L1 inhibitor in a phase Ib clinical trial showed favorable efficacy in previously treated patients with advanced TNBC with a manageable safety profile; Combination chemotherapy for treatment of metastatic TNBC achieves efficacy and is safe and controlled in phase II clinical trial studies.	(107, 108)
Gefitinib	EGFR	Efficacy achieved in combination with neoadjuvant chemotherapy in TNBC patients in a randomized phase II clinical trial study, but the trial was terminated due to toxic events.	(109)
Erlotinib	EGFR	Combined bevacizumab maintenance therapy reduces tumor load in most patients in a phase II clinical trial study.	(110)
Ruxolitinib	JAK2	In a phase II clinical trial study, treatment of TNBC as a single agent did not meet efficacy endpoints; Combination capecitabine has no benefit over capecitabine alone for TNBC in a phase II clinical trial study; Combined PTX is better than PTX alone for TNBC in a phase I clinical trial study.	(111–113)

Gefitinib, a small molecule EGFR inhibitor, along with neoadjuvant chemotherapy, showed a higher pathological complete response rate in a Phase II clinical trial for patients with TNBC, especially in the chemotherapy and Gefitinib combination group. However, it is critical to note that patients receiving Gefitinib exhibited a higher incidence of toxic reactions, consequently leading to the discontinuation of the trial for those patients (109). In another Phase II clinical trial, Erlotinib, a small molecule EGFR inhibitor, was evaluated for its efficacy in treating metastatic TNBC. Patients in this trial initially received treatment with albumin-bound PTX combined with bevacizumab, followed by a maintenance regimen comprising both bevacizumab and Erlotinib. Notably, a significant proportion of participants in this trial exhibited partial tumor responses (110).

Research on RTK inhibitors in TNBC is expanding to include natural products such as Salidroside extracted from Rhodiola (**Table 2**). Preclinical studies have shown that Salidroside inhibits phosphorylation signaling pathways of EGFR/JAK2/STAT3, thereby impacting TNBC cell viability by binding to EGFR. Salidroside’s therapeutic potential is highlighted by its selective efficacy, demonstrating minimal toxicity in normal breast epithelial cells (114). Doxazosin, primarily known as a vasodilator, has a dual-target mechanism, binding to cellular mesenchymal-epithelial transition factor (c-MET) and EGFR. This binding results in the inhibition of JAK2/STAT3 phosphorylation signaling. Research has demonstrated that doxazosin significantly affects TNBC cell proliferation, invasion, and migration, supported by *in vitro* and *in vivo* evidence. The efficacy of doxazosin in curbing TNBC lung metastasis was further substantiated through a mouse lung metastasis model (115).

**Table 2:** Targeting RTKs to modulate the JAK2/STAT3 signaling pathway in preclinical studies for the treatment of TNBC.

Compd.	Target	*In Vivo* Or *In Vitro*	Citation
Salidroside	EGFR	*In vitro*	(114)
Doxazosin	EGFR/c-MET	*In vitro* and *in vivo*	(115)
Magnolol	EGFR	*In vitro*	(116)
4-(adamantan-1-yl)-2-(3-(2,4-dichlorophenyl)-5-phenyl-4,5-dihydro-1*H*-pyrazol-1-yl)thiazole (APP)	EGFR	*In vitro*	(117)
Demethoxycurcumin	EGFR	*In vitro*	(118)
Morin	EGFR	*In vitro*	(119, 120)
Primaquine	EGFR	*In vitro* and *in vivo*	(121)
Centipeda minima Extract (CME)	EGFR	*In vitro* and *in vivo*	(122)
CAPE-*p*NO2	EGFR	*In vitro* and *in vivo*	(123)
Phospho-aspirin-2 (PA-2)	EGFR	*In vitro* and *in vivo*	(124)
Deguelin	EGFR/c-MET	*In vitro* and *in vivo*	(125, 126)
Picrasidine G	EGFR	*In vitro*	(127)
Regorafenib	VEGFR/PDGFR	*In vitro* and *in vivo*	(128)
Bazedoxifene	GP130	*In vitro* and *in vivo*	(129, 130)
Raloxifene	GP130	*In vitro*	(131)
EC359	LIFR	*In vitro* and *in vivo*	(34, 132)
Chikusetsusaponin IVa Butyl Ester (CS-IVa-Be)	IL-6R	*In vitro*	(133)

Magnolol, a multifunctional lignan compound derived from the traditional Chinese herb Houpo, has demonstrated notable anti-cancer properties against TNBC. *In vitro* studies reveal that Magnolol effectively reduces the viability of TNBC cells. This inhibitory effect is primarily attributed to the suppression of phosphorylation signaling in the EGFR/JAK2/STAT3 pathway (116). The lead compound APP has also shown promising results in TNBC treatment. *In vitro* analyses indicate that APP induces apoptosis in TNBC cells. This apoptotic effect is mediated through the inhibition of EGFR/JAK2/STAT3 phosphorylation signaling, coupled with the regulation of apoptotic proteins (117). Demethoxycurcumin (DMC), a principal variant of curcumin predominantly found in the rhizomes of turmeric, has garnered attention in the context of TNBC research. *In vitro* studies have illuminated its potential in modulating TNBC cell viability. DMC achieves this by inhibiting EGFR protein expression levels. Furthermore, its mechanism involves the inhibition of specific phosphatases, thereby sustaining EGFR activation. This suggests that DMC’s influence on TNBC cells might result from its regulation of multiple signaling pathways (118). Morin, a flavonoid compound derived from plants, has shown potential in the treatment of TNBC. Studies indicate that Morin, particularly when used in conjunction with doxorubicin, promotes apoptosis in TNBC cells. This synergistic effect is attributed to the inhibition of EGFR/STAT3 phosphorylation signaling (119, 120).

Primaquine, an antimalarial drug, has been observed to inhibit TNBC cell viability and migration *in vitro*. This inhibition is linked to the suppression of EGFR/STAT3 phosphorylation signaling. However, the specific mechanisms by which Primaquine impedes TNBC growth *in vivo* remain to be explored further (121). Additionally, Centipeda minima Extract (CME), an extract from the Centipeda minima, has demonstrated efficacy in regulating TNBC cell behavior by modulating the phosphorylation signaling of multiple pathways, notably the STAT3 pathway. This modulation occurs through the inhibition of EGFR expression, thereby promoting apoptosis in TNBC cells (122). CAPE-pNO2 has also been identified as a potent inhibitor of proliferation and migration in TNBC by suppressing EGFR phosphorylation and the regulation of STAT3 and AKT phosphorylation signaling. This dual effect has been observed in both *in vitro* and *in vivo* studies (123).

Similarly, PA-2, another compound under investigation, has demonstrated its ability to promote apoptosis in TNBC cells. It achieves this through the inhibition of EGFR phosphorylation and by modulating the phosphorylation signaling of the PI3K/AKT and STAT3 pathways (124). Deguelin also contributes to this growing field of TNBC therapeutics. It influences the expression of both EGFR and c-Met, leading to the downregulation of phosphorylation signaling across several pathways, including STAT3, AKT, ERK, and NFκB. This comprehensive action results in a marked impact on the viability of TNBC cells (125, 126). Picrasidine G, a naturally derived dimeric alkaloid, has shown efficacy in inhibiting the vitality of TNBC cells *in vitro* by suppressing the EGFR/STAT3 phosphorylation signaling pathway (127). Regorafenib, another compound under study, exerts its anti-cancer effects by inhibiting key receptors such as VEGFR and PDGFR. This inhibition impacts the STAT3 phosphorylation signaling (128).

Bazedoxifene presents a different angle in TNBC treatment. By targeting GP130, it influences the STAT3 phosphorylation signaling pathway. This strategic inhibition inhibits TNBC both *in vitro* and *in vivo (* 129, 130). Raloxifene, a compound known for its influence on the GP130 receptor, has been shown to inhibit the vitality of TNBC cells *in vitro*. This effect is achieved through the modulation of the STAT3 phosphorylation signaling pathway (131). EC359, another promising agent, binds to the leukemia inhibitory factor receptor (LIFR), inhibiting the LIFR/STAT3 phosphorylation signaling, thereby curbing TNBC proliferation both *in vitro* and *in vivo (* 34, 132). Similarly, CS-IVa-Be targets cancer cells through the inhibition of IL-6R, impacting the JAK2/STAT3 phosphorylation signaling pathway. This specific action has been observed to inhibit TNBC *in vitro (* 133).

In summary, RTK inhibitors can inhibit the signaling of the JAK2/STAT3 pathway. However, due to the activation of bypass pathways, clinical trial results suggest that combining these inhibitors with chemotherapy may be more beneficial for patients with TNBC.

#### 5.1.2 JAK2 inhibitors

Directly targeting JAK2 emerges as a strategic approach for modulating the JAK2/STAT3 signaling pathway in TNBC treatment. However, to date, JAK2 inhibitors have not received FDA approval for use in TNBC therapy. Ruxolitinib, a JAK1/JAK2 inhibitor, is being evaluated for its clinical efficacy in treating TNBC in several clinical trials. In a Phase II clinical trial, Ruxolitinib monotherapy did not meet its efficacy endpoint for TNBC treatment (111). Further research explored the potential of Ruxolitinib in combination with chemotherapy drugs. While combining Ruxolitinib with Capecitabine did not enhance overall survival, another trial pairing it with PTX showed improved clinical efficacy, outperforming PTX monotherapy (112, 113) (**Table 1**).

In preclinical TNBC studies, several small molecules exhibit promise as JAK2 inhibitors (**Table 3**). For instance, Glyceryl Trinitrate (GTN), a vasodilator, inhibits STAT3 activation by blocking JAK2 phosphorylation, suppressing TNBC cell viability (134). Additionally, a range of compounds, including Withaferin A (WA) (135), Naphtho[1,2-b]furan-4,5-dione (NFD) (136), Ganoderic acid A (GA-A) (137), Methylseleninic Acid (MSA) (138), and AZD1480 (139), have been identified to inhibit the phosphorylation and signal transduction of the JAK2/STAT3 pathway, reducing the viability of TNBC cells. Recent research findings highlight the efficacy of JAK2 small molecule inhibitors in TNBC. For instance, the JAK2 inhibitor AG490 has been shown to reduce TNBC cell viability by modulating the phosphorylation and signal transduction of STAT3 and AKT (140, 141). Additionally, 3-Deoxy-2β,16-dihydroxynagilactone E (B6) interacts with the FERM-SH2 domain of JAK2, inhibiting downstream STAT3 phosphorylation and reducing TNBC cell viability (142). Another study highlights the effectiveness of ECN in suppressing TNBC cell viability by targeting the JAK2/STAT3 signaling pathway. ECN has also demonstrated the ability to inhibit TNBC tumor growth *in vivo (* 143).

**Table 3:** Targeting the JAK2 for TNBC in preclinical studies.

Compd.	Target	*In Vivo* Or *In Vitro*	Citation
Glyceryl Trinitrate (GTN)	JAK2	*In vitro* and *in vivo*	(134)
Withaferin A (WA)	JAK2	*In vitro*	(135)
Naphtho[1,2-b]furan-4,5-dione (NFD)	JAK2	*In vitro*	(136)
Ganoderic acid A (GA-A)	JAK2	*In vitro*	(137)
Methylseleninic Acid (MSA)	JAK2	*In vitro* and *in vivo*	(138)
AZD1480	JAK2	*In vitro*	(139)
AG490	JAK2	*In vitro*	(140, 141)
3-Deoxy-2β,16-dihydroxynagilactone E (B6)	JAK2	*In vitro*	(142)
7β-(3-Ethyl-*cis*-crotonoyloxy)-1α-(2-methylbutyryloxy)-3,14-dehydro-Z-notonipetranone (ECN)	JAK2	*In vitro* and *in vivo*	(143)
Chloroquine	JAK2	*In vitro* and *in vivo*	(95)
Silibinin	JAK2	*In vitro*	(144, 145)
Piperlongumine	JAK2	*In vitro* and *in vivo*	(146)
Hydroxyzine	JAK2	*In vitro*	(147)

Chloroquine enhances PTX therapeutic efficacy in TNBC by inhibiting JAK2/STAT3 pathway phosphorylation, impacting autophagy processes (95). Additionally, Silibinin suppresses TNBC cell invasive and migratory capabilities *in vitro* by downregulating JAK2/STAT3 pathway phosphorylation (144, 145). Piperlongumine, a bioactive alkaloid known for its antioxidant and anti-tumor properties, has been found to inhibit TNBC cell proliferation and migration by inhibiting JAK2/STAT3 pathway phosphorylation (146). Similarly, Hydroxyzine, primarily recognized as a histamine H1 receptor antagonist, has demonstrated the capability to induce apoptosis in TNBC cells through the inhibition of JAK2/STAT3 phosphorylation (147).

JAK2 inhibitors have shown significant efficacy in inhibiting TNBC *in vitro*. Clinical trial data *in vivo* also suggest that a combination of these inhibitors with the chemotherapy drug paclitaxel could be a promising therapeutic approach for TNBC. However, concerns about the safety of JAK2 inhibitors, as evidenced by FDA warnings, underscores the necessity for alternative therapeutic strategies targeting the JAK2/STAT3 pathway.

#### 5.1.3 STAT3 inhibitor

Several STAT3 small molecule inhibitors have been reported in preclinical studies for the treatment of TNBC (**Table 4**). The therapeutic strategy to inhibit STAT3 involves targeting multiple stages of its functional cycle, including phosphorylation, dimerization, nuclear translocation, and DNA binding activities. This approach leverages the nuclear translocation signal of STAT3.

**Table 4:** Targeting STAT3 for TNBC in preclinical studies.

Compd.	Target	*In Vivo* Or *In Vitro*	Citation
Stattic	STAT3	*In vitro*	(148, 149)
STA-21	STAT3	*In vitro*	(150)
FLLL31	STAT3	*In vitro*	(151)
FLLL32	STAT3	*In vitro* and *in vivo*	(151)
Pyrrolidinesulphonylaryl molecules (6a)	STAT3	*In vitro*	(152)
LLL12	STAT3	*In vitro* and *in vivo*	(153)
LLL12B	STAT3	*In vitro* and *in vivo*	(15)
Naringenin	STAT3	*In vitro*	(154)
S3I-201	STAT3	*In vitro*	(155)
Napabucasin	STAT3	*In vitro*	(156)
Coumarin-benzothiophene1, 1-dioxide conjugates compound(7a)	STAT3	*In vitro* and *in vivo*	(157)
SLSI-1216	STAT3	*In vitro*	(158)
H182	STAT3	*In vitro* and *in vivo*	(159)
SMY002	STAT3	*In vitro* and *in vivo*	(160)
MC0704	STAT3	*In vitro* and *in vivo*	(161)
ZSW	STAT3	*In vitro* and *in vivo*	(162)
Acetyl-cinobufagin	STAT3	*In vitro* and *in vivo*	(163)
Arctigenin	STAT3	*In vitro* and *in vivo*	(164)
KYZ3	STAT3	*In vitro* and *in vivo*	(165)
Dihydrotanshinone	STAT3	*In vitro* and *in vivo*	(166)
DT-13	STAT3	*In vitro* and *in vivo*	(167)
Cucurbitacin E	STAT3	*In vitro*	(168–170)
Niclosamide	STAT3	*In vitro* and *in vivo*	(171–173)
SG-1709	STAT3	*In vitro*	(174)
SG-1721	STAT3	*In vitro* and *in vivo*	(174)
Nifuroxazide	STAT3	*In vitro* and *in vivo*	(175, 176)
LLY17	STAT3	*In vitro* and *in vivo*	(177)
6Br-6a	STAT3	*In vitro* and *in vivo*	(178)
Pyrimethamine	STAT3	*In vitro* and *in vivo*	(179, 180)
Pectolinarigenin	STAT3	*In vitro* and *in vivo*	(181)
Flubendazole	STAT3	*In vitro* and *in vivo*	(182, 183)
Eupalinolide J	STAT3	*In vitro*	(184, 185)
Betulinic acid	STAT3	*In vitro* and *in vivo*	(186)
Carfilzomib	STAT3	*In vitro* and *in vivo*	(187)
WP1066	STAT3	*In vitro*	(188)
Rhus coriaria extract	STAT3	*In vitro* and *in vivo*	(189)
FZU-03,010	STAT3	*In vitro*	(190)
Disulfiram	STAT3	*In vitro*	(191)
Schisandrin B	STAT3	*In vitro* and *in vivo*	(192)
Osthole	STAT3	*In vitro* and *in vivo*	(193)
Brevilin A	STAT3	*In vitro* and *in vivo*	(194)
Arnicolide D	STAT3	*In vitro* and *in vivo*	(195)
Eucannabinolide	STAT3	*In vitro* and *in vivo*	(196)
Pulvomycin	STAT3	*In vitro* and *in vivo*	(197)
R001	STAT3	*In vitro* and *in vivo*	(198)
Salinomycin	STAT3	*In vitro*	(199, 200)
Ethanolic extract of Origanum syriacum	STAT3	*In vitro*	(201)
Apigenin	STAT3	*In vitro* and *in vivo*	(202)
AG-014699	STAT3	*In vitro*	(203)

Stattic, a non-peptidic small molecule, has demonstrated notable anti-TNBC effects by selectively targeting STAT3, inhibiting its activation, dimerization, and nuclear translocation. This inhibition is facilitated through Stattic’s binding to the SH2 functional domain of STAT3 (148, 149). Similarly, STA-21, another small molecule inhibitor, induces apoptosis in TNBC cells by inhibiting DNA binding activity and dimerization of STAT3 (150). FLLL31 and FLLL32, derivatives of curcumin, have been identified as selective inhibitors of STAT3. They achieve this by binding to the SH2 functional domain of STAT3, thereby inhibiting its phosphorylation and DNA binding activities. Notably, these compounds have shown potential in synergistically inhibiting TNBC cell proliferation when combined with doxorubicin. *In vivo* studies further indicate that FLLL32 can effectively suppress TNBC growth by downregulating STAT3 phosphorylation levels (151). Pyrrolidine sulfonamide derivative **6a** selectively inhibits STAT3 activation at phosphorylation and transcription levels, reducing TNBC cell viability in response to IL-6 stimulation (152). LLL12, a non-peptidic, cell-permeable small molecule, selectively targets STAT3 by inhibiting its DNA binding activity and phosphorylation through SH2 domain binding. It induces apoptosis in TNBC cells and suppresses TNBC growth *in vivo* by downregulating STAT3 phosphorylation levels (153). LLL12B, a prodrug of LLL12, is activated in the tumor microenvironment by tumor-associated plasmin, which cleaves its aminoformate bond to release active LLL12. LLL12B exhibits improved pharmacokinetic properties compared to its parent compound, LLL12. However, additional research is required to fully elucidate the comparative *in vivo* and *in vitro* pharmacology of these compounds, particularly their respective abilities to bind to STAT3 (15).

Naringenin, a naturally occurring compound, reduces TNBC cell viability by binding to the SH2 domain of STAT3, suppressing STAT3 phosphorylation. In combination with cyclophosphamide, naringenin has demonstrated enhanced efficacy in inducing apoptosis in TNBC cells (154). S3I-201, a selective STAT3 inhibitor probe, targets the SH2 functional domain of STAT3, inhibiting its DNA binding activity and dimerization. *In vitro* studies have revealed that S3I-201 significantly diminishes the TNBC cell viability and inhibits tumor growth by reducing STAT3 phosphorylation (155). Napabucasin, a targeted therapeutic agent, selectively inhibits the DNA binding activity and phosphorylation of STAT3 by binding to its SH2 functional domain. *In vitro* studies have demonstrated Napabucasin’s capability to reduce TNBC cell viability (156). Additionally, a series of compounds, such as 7a (157), SLSI-1216 (158), H182 (159), SMY002 (160), MC0704 (161), ZSW (162), and Acetyl-cinobufagin (163), have been identified to selectively inhibit STAT3 phosphorylation by binding to its SH2 domain and suppressing TNBC cell viability *in vitro*. Arctigenin, a bioactive lignan isolated from the seeds of Arctium lappa, inhibits STAT3 in TNBC cells by binding to its SH2 domain, thereby disrupting hydrogen bond connections between DNA and STAT3. This disruption prevents STAT3’s binding to genomic DNA, effectively reducing TNBC cell viability (164). Similarly, KYZ3, a derivative of cryptotanshinone, binds to the SH2 domain of STAT3, inhibiting its DNA binding activity and phosphorylation, leading to decreased TNBC cell viability *in vitro (* 165). Research has identified a wide array of small molecules that exhibit the potential to inhibit TNBC cell viability. This includes Dihydrotanshinone (166), DT-13 (167), Cucurbitacin E (168–170), Niclosamide (171–173), SG-1709 (174), SG-1721 (174), Nifuroxazide (175, 176), LLY17 (177), 6Br-6a (178), Pyrimethamine (179, 180), Pectolinarigenin (181), Flubendazole (182, 183), Eupalinolide J (184, 185), Betulinic acid (186), Carfilzomib (187), WP1066 (188), Rhus coriaria extract (189), FZU-03,010 (190), Disulfiram (191), Schisandrin B (192), Osthole (193), Brevilin A (194), Arnicolide D (195), Eucannabinolide (196), Pulvomycin (197), R001 (198), Salinomycin (199, 200), the ethanolic extract of origanum syriacum (201), Apigenin (202), and AG-014699 (203). This inhibition is attributed to their ability to suppress STAT3 phosphorylation. However, direct evidence demonstrating their binding to STAT3 is currently lacking.

While preclinical studies have identified various small molecule compounds as potential STAT3 inhibitors, TTI-101 stands out as the sole compound advancing into Phase I clinical trials. Various groups of researchers are investigating the efficacy and safety of TTI-101 in patients with advanced breast cancer and those with inoperable solid tumors.

#### 5.1.4 Adverse effects of JAK2/STAT3 pathway inhibition

Because the biological processes of normal cells also depend on the JAK2/STAT3 pathway, the long-term use of JAK2/STAT3 pathway inhibitors has certain toxic side effects. JAK2 inhibitors inevitably inhibit the normal hematopoietic function of the body, which can cause anemia, thrombocytopenia, and other adverse effects (including dizziness, headache, abdominal pain, diarrhea, and the secondary tumor).

Studies have shown that anemia and thrombocytopenia are the most common hematologic adverse effects when JAK2 inhibitors are used to treat myelofibrosis (204–210); in another study of Ruxolitinib as a drug treatment for true erythrocytosis, headache and diarrhea were the most common non-hematologic adverse effects (211); the immunosuppressive effects of JAK2 inhibitors are important in inducing infections, and in the JUMP study, the most common infection was pneumonia, followed by urinary tract infections and nasopharyngitis (205), and another study showed that 30 of 31 patients treated with Ruxolitinib developed infections, including several opportunistic infections (212); although JAK inhibitors can be used to treat hematologic cancers and inflammatory diseases, during treatment with these drugs, studies have found that some patients suffer from lymphomas and other malignancies, with a statistically significant 16-fold increase in the risk of B-cell malignancies in patients with myeloproliferative neoplasms treated with JAK1/2 inhibitors, and skin cancers being the most common secondary tumor (213); in addition to these symptoms, Ruxolitinib can also cause other adverse reactions such as abdominal pain, drowsiness, acute renal failure (211), and even some studies have reported that patients have died from cardiac arrest (204).

Since the JAK family mediates signaling of multiple cytokines and different receptors are associated with different JAKs, and comprehensive inhibition of the JAK family can result in a variety of side effects, the design and development of new targeted JAK2 inhibitors could provide a solution to these adverse effects.

### 5.2 Event-driven mode: application of PROTAC molecules based on ubiquitin-mediated protein degradation in TNBC

The regulation of the JAK2/STAT3 signaling pathway can be strategically achieved through the targeted degradation of the JAK2 protein employing PROTACs. These molecular constructs consist of a linker connecting two ligands, with one ligand binding to the target JAK2 protein and the other engaging with the ubiquitin E3 ligase. This dual binding facilitates the formation of a ternary complex, bringing the JAK2 protein and E3 ligase into close proximity. Subsequently, the target JAK2 protein undergoes ubiquitination, marking it for recognition by the proteasome system. This leads to the proteasomal degradation of JAK2 into peptide fragments, effectively nullifying its protein activity (214). Recent studies underscore the promising role of PROTAC molecules in TNBC treatment. MZ1, a small molecule PROTAC, targets BRD4 protein for degradation. Compared to JQ1, a conventional inhibitor targeting the protein domain, MZ1 exhibits superior anti-TNBC activity both *in vitro* and *in vivo*, which is attributed to its specific action in targeting BRD4 protein degradation (215). Another notable PROTAC molecule, NN3, is designed to target PARP1 protein degradation. Experimental findings indicate that NN3 demonstrates effective anti-TNBC activity *in vitro* and *in vivo*. Remarkably, NN3 retains its efficiency in degrading PARP1 protein even in the presence of point mutations, further underscoring its potential as an anti-tumor agent (216). Emerging research sheds light on the efficacy of PROTAC molecule 6n, designed to target the degradation of the AXL protein. Demonstrating a significant advantage over traditional AXL kinase inhibitors, 6n has shown superior anti-TNBC activity *in vitro* and *in vivo (* 217). Similarly, YX-02-030, a PROTAC molecule targeting the MDM2 protein degradation, exhibits enhanced anti-tumor activity compared to specific MDM2 inhibitors. Notably, YX-02-030 achieves this therapeutic efficacy without causing harm to normal cells (218). TEP, a PROTAC molecule engineered to target c-Myc protein degradation, effectively inhibits the proliferation of TNBC cells by facilitating the specific degradation of the endogenous c-Myc/Max complex. Additionally, TEP enhances the sensitivity of TNBC cells to palbociclib, a cyclin-dependent kinase inhibitor (219). Another PROTAC molecule, CT-4, designed to target HDAC8 protein degradation, promotes apoptosis in TNBC cells through the targeted degradation of HDAC8 protein (220). The small molecule compound A4, a PROTAC developed based on DCAF16, specifically targets CDK4/6 protein degradation. Research demonstrates that A4 exhibits potent inhibitory activity against CDK4/6, offering a favorable safety profile in normal cells, which is considered superior to the established CDK4/6 inhibitor, palbociclib (221). The small molecule compounds 7f and PP-C8, which also function as PROTAC molecules, also target CDK12/13 degradation. Studies have indicated that these compounds effectively reduce TNBC cell viability by inhibiting the expression of CDK12/13 (222, 223). PROTAC molecules MS8815 and U3i, designed to target EZH2 protein degradation, induce ubiquitination and subsequent proteasome-dependent degradation of EZH2, effectively inhibiting TNBC cell growth (224, 225). Similarly, androgen receptor (AR)-PROTAC has shown efficacy in targeting AR-positive TNBC cells by mediating the ubiquitination and degradation of the AR, thereby inhibiting cell growth (226). Furthermore, C8, a PROTAC molecule developed based on the PARP1/2 inhibitor Olaparib, exhibits promising therapeutic potential against TNBC. It promotes PARP2 protein degradation, demonstrating effectiveness *in vitro* and *in vivo (* 227).

Currently, PROTAC small molecules targeting the degradation of JAK2 protein have been reported. However, research indicates that the E3 ligase CUL5 mediates JAK2 protein degradation (228). Developing PROTAC small molecules that facilitate the binding of JAK2 protein to the E3 ligase CUL5 could be a feasible strategy for treating TNBC.

## 6 Conclusion

The JAK2/STAT3 signaling pathway, activated by cytokines, is central in governing fundamental cellular processes, including growth, differentiation, apoptosis, and immune responses. In TNBC, excessive activation of this pathway contributes to immune evasion by TNBC cells. This aberrant activation promotes tumor growth, facilitates metastasis, and develops drug resistance in TNBC. Therefore, the strategic therapeutic targeting of the JAK2/STAT3 signaling pathway emerges as a promising strategy for the effective treatment of TNBC. The JAK2/STAT3 signaling pathway presents multiple targets for therapeutic intervention in TNBC. Inhibition strategies focusing on RTKs, JAK2, and STAT3 effectively suppresses TNBC cell growth. Despite these promising results, clinical trials of inhibitors that bind to the active sites of these proteins have encountered challenges. These limitations can be attributed to several factors, including the activation of compensatory bypass pathways, overexpression of target proteins, emergence of point mutations within these targets, and the heightened expression of competitive ligands. Unlike conventional inhibitors, PROTAC molecules do not rely on sustained binding to the target protein to exert their inhibitory effect. This unique characteristic enables them to remain effective even in the presence of mutations in the protein’s active binding site. A key advantage of PROTACs lies in their catalytic mechanism; following the facilitation of ubiquitination and degradation of the target protein, PROTAC molecules can be recycled. This recycling ability potentially allows for lower drug dosages, enhancing both the safety profile and therapeutic potential of these molecules. Consequently, employing PROTACs to target the JAK2/STAT3 pathway emerges as an exceptionally promising strategy for TNBC treatment. By developing PROTACs that specifically target JAK2 protein degradation, not only is TNBC growth inhibited through the downregulation of the JAK2/STAT3 pathway, but the typical toxic side effects associated with traditional JAK2 inhibitors are also likely to be mitigated.

## Author’s note

During the preparation of this work authors did not used any AI tool/service, and takes full responsibility for the content of the publication.

## Author contributions

LL: Conceptualization, Resources, Writing – original draft. XF: Conceptualization, Resources, Writing – original draft. LC: Formal Analysis, Project administration, Writing – original draft. LY: Conceptualization, Supervision, Writing – review & editing. XL: Conceptualization, Supervision, Writing – review & editing.

## References

[1] Sung H Ferlay J Siegel RL Laversanne M Soerjomataram I Jemal A. Global cancer statistics 2020: GLOBOCAN estimates of incidence and mortality worldwide for 36 cancers in 185 countries. CA Cancer J Clin. (2021) 71:209–49. doi: 10.3322/caac.21660 33538338

[2] Abdelwahab YA. Male breast cancer: epidemiology and risk factors. Semin Oncol. (2017) 44:267–72. doi: 10.1053/j.seminoncol.2017.11.002 29526255

[3] Geyer FC Pareja F Weigelt B Rakha E Ellis IO Schnitt SJ. The spectrum of triple-negative breast disease: high- and low-grade lesions. Am J Pathol. (2017) 187:2139–51. doi: 10.1016/j.ajpath.2017.03.016 PMC5809519 28736315

[4] Ellington TD Miller JW Henley SJ Wilson RJ Wu M Richardson LC. Trends in breast cancer incidence, by race, ethnicity, and age among women aged >/=20 years - United States, 1999-2018. Mmwr Morb Mortal Wkly Rep. (2022) 71:43–7. doi: 10.15585/mmwr.mm7102a2 PMC8757618 35025856

[5] Burguin A Diorio C Durocher F. Breast cancer treatments: updates and new challenges. J Pers Med. (2021) 11:808. doi: 10.3390/jpm11080808 34442452 PMC8399130

[6] Zarrin AA Bao K Lupardus P Vucic D. Kinase inhibition in autoimmunity and inflammation. Nat Rev Drug Discovery. (2021) 20:39–63. doi: 10.1038/s41573-020-0082-8 33077936 PMC7569567

[7] Banerjee S Biehl A Gadina M Hasni S Schwartz DM. JAK-STAT signaling as a target for inflammatory and autoimmune diseases: current and future prospects. Drugs. (2017) 77:521–46. doi: 10.1007/s40265-017-0701-9 PMC7102286 28255960

[8] Jiang YZ Ma D Suo C Shi J Xue M Hu X. Genomic and transcriptomic landscape of triple-negative breast cancers: subtypes and treatment strategies. Cancer Cell. (2019) 35:428–40. doi: 10.1016/j.ccell.2019.02.001 30853353

[9] Zheng Q Han L Dong Y Tian J Huang W Liu Z. JAK2/STAT3 targeted therapy suppresses tumor invasion via disruption of the EGFRvIII/JAK2/STAT3 axis and associated focal adhesion in EGFRvIII-expressing glioblastoma. Neuro Oncol. (2014) 16:1229–43. doi: 10.1093/neuonc/nou046 PMC4136898 24861878

[10] Xu J Zhang L Li N Dai J Zhang R Yao F. Etomidate elicits anti-tumor capacity by disrupting the JAK2/STAT3 signaling pathway in hepatocellular carcinoma. Cancer Lett. (2023) 552:215970. doi: 10.1016/j.canlet.2022.215970 36265652

[11] Liang Q Gong M Zou JH Luo MY Jiang LL Wang C. A phosphoglycerate mutase 1 allosteric inhibitor overcomes drug resistance to EGFR-targeted therapy via disrupting IL-6/JAK2/STAT3 signaling pathway in lung adenocarcinoma. Drug Resist Update. (2023) 68:100957. doi: 10.1016/j.drup.2023.100957 36990047

[12] Zhang L Yang Z Ma A Qu Y Xia S Xu D. Growth arrest and DNA damage 45G down-regulation contributes to Janus kinase/signal transducer and activator of transcription 3 activation and cellular senescence evasion in hepatocellular carcinoma. Hepatology. (2014) 59:178–89. doi: 10.1002/hep.26628 23897841

[13] Chang R Song L Xu Y Wu Y Dai C Wang X. Loss of Wwox drives metastasis in triple-negative breast cancer by JAK2/STAT3 axis. Nat Commun. (2018) 9:3486. doi: 10.1038/s41467-018-05852-8 30154439 PMC6113304

[14] Chen M Pockaj B Andreozzi M Barrett MT Krishna S Eaton S. JAK2 and PD-L1 amplification enhance the dynamic expression of PD-L1 in triple-negative breast cancer. Clin Breast Cancer. (2018) 18:e1205–15. doi: 10.1016/j.clbc.2018.05.006 29933930

[15] Pan L Chen X Rassool FV Li C Lin J. LLL12B, a novel small-molecule STAT3 inhibitor, induces apoptosis and suppresses cell migration and tumor growth in triple-negative breast cancer cells. Biomedicines. (2022) 10:2003. doi: 10.3390/biomedicines10082003 36009550 PMC9405793

[16] Wang Y Cheng Z Xu J Lai M Liu L Zuo M. Fat mass and obesity-associated protein (FTO) mediates signal transducer and activator of transcription 3 (STAT3)-drived resistance of breast cancer to doxorubicin. Bioengineered. (2021) 12:1874–89. doi: 10.1080/21655979.2021.1924544 PMC8806322 34076564

[17] Jiao K Zhen J Wu M Teng M Yang K Zhou Q. 27-Hydroxycholesterol-induced EndMT acts via STAT3 signaling to promote breast cancer cell migration by altering the tumor microenvironment. Cancer Biol Med. (2020) 17:88–100. doi: 10.20892/j.issn.2095-3941.2019.0262 32296578 PMC7142833

[18] Lim JH. Inhibition of the interleukin-11-STAT3 axis attenuates hypoxia-induced migration and invasion in MDA-MB-231 breast cancer cells. Korean J Physiol Pharmacol. (2014) 18:391–96. doi: 10.4196/kjpp.2014.18.5.391 PMC4211122 25352758

[19] Falamarzian A Aliabadi HM Molavi O Seubert JM Lai R Uludag H. Effective down-regulation of signal transducer and activator of transcription 3 (STAT3) by polyplexes of siRNA and lipid-substituted polyethyleneimine for sensitization of breast tumor cells to conventional chemotherapy. J BioMed Mater Res A. (2014) 102:3216–28. doi: 10.1002/jbm.a.34992 24167124

[20] Pawlus MR Wang L Hu CJ. STAT3 and HIF1alpha cooperatively activate HIF1 target genes in MDA-MB-231 and RCC4 cells. Oncogene. (2014) 33:1670–79. doi: 10.1038/onc.2013.115 PMC3868635 23604114

[21] Deng XS Wang S Deng A Liu B Edgerton SM Lind SE. Metformin targets Stat3 to inhibit cell growth and induce apoptosis in triple-negative breast cancers. Cell Cycle. (2012) 11:367–76. doi: 10.4161/cc.11.2.18813 22189713

[22] Tripolt S Neubauer HA Knab VM Elmer DP Aberger F Moriggl R. Opioids drive breast cancer metastasis through the delta-opioid receptor and oncogenic STAT3. Neoplasia. (2021) 23:270–79. doi: 10.1016/j.neo.2020.12.011 PMC7815495 33465556

[23] Zammarchi F de StanChina E Bournazou E Supakorndej T Martires K Riedel E. Antitumorigenic potential of STAT3 alternative splicing modulation. Proc Natl Acad Sci U.S.A. (2011) 108:17779–84. doi: 10.1073/pnas.1108482108 PMC3203802 22006329

[24] Ling X Arlinghaus RB. Knockdown of STAT3 expression by RNA interference inhibits the induction of breast tumors in immunocompetent mice. Cancer Res. (2005) 65:2532–36. doi: 10.1158/0008-5472.CAN-04-2425 15805244

[25] Wang S Liang K Hu Q Li P Song J Yang Y. JAK2-binding long noncoding RNA promotes breast cancer brain metastasis. J Clin Invest. (2017) 127:4498–515. doi: 10.1172/JCI91553 PMC5707156 29130936

[26] Han J Yun J Quan M Kang W Jung JG Heo W. JAK2 regulates paclitaxel resistance in triple negative breast cancers. J Mol Med (Berl). (2021) 99:1783–95. doi: 10.1007/s00109-021-02138-3 34626199

[27] Hubbard SR Till JH. Protein tyrosine kinase structure and function. Annu Rev Biochem. (2000) 69:373–98. doi: 10.1146/annurev.biochem.69.1.373 10966463

[28] Zhao D Pan C Sun J Gilbert C Drews-Elger K Azzam DJ. VEGF drives cancer-initiating stem cells through VEGFR-2/Stat3 signaling to upregulate Myc and Sox2. Oncogene. (2015) 34:3107–19. doi: 10.1038/onc.2014.257 25151964

[29] Wintheiser GA Silberstein P. Physiology, tyrosine kinase receptors. In StatPearls. StatPearls Publishing (2024). 30860767

[30] Zhang M Zhang X Zhao S Wang Y Di W Zhao G. Prognostic value of survivin and EGFR protein expression in triple-negative breast cancer (TNBC) patients. Target Oncol. (2014) 9:349–57. doi: 10.1007/s11523-013-0300-y 24233638

[31] Bustamante-Marin X Devlin KL McDonell SB Dave O Merlino JL Grindstaff EJ. Regulation of IGF1R by microRNA-15b contributes to the anticancer effects of calorie restriction in a murine C3-TAg model of triple-negative breast cancer. Cancers (Basel). (2023) 15:4320. doi: 10.3390/cancers15174320 37686596 PMC10486801

[32] D’Ippolito E Plantamura I Bongiovanni L Casalini P Baroni S Piovan C. miR-9 and miR-200 regulate PDGFRbeta-mediated endothelial differentiation of tumor cells in triple-negative breast cancer. Cancer Res. (2016) 76:5562–72. doi: 10.1158/0008-5472.CAN-16-0140 27402080

[33] Wu Y Yi Z Li J Wei Y Feng R Liu J. FGFR blockade boosts T cell infiltration into triple-negative breast cancer by regulating cancer-associated fibroblasts. Theranostics. (2022) 12:4564–80. doi: 10.7150/thno.68972 PMC9254240 35832090

[34] Li M Viswanadhapalli S Santhamma B Pratap UP Luo Y Liu J. LIFR inhibition enhances the therapeutic efficacy of HDAC inhibitors in triple negative breast cancer. Commun Biol. (2021) 4:1235. doi: 10.1038/s42003-021-02741-7 34716410 PMC8556368

[35] Weng YS Tseng HY Chen YA Shen PC Al HA Chen LM. MCT-1/miR-34a/IL-6/IL-6R signaling axis promotes EMT progression, cancer stemness and M2 macrophage polarization in triple-negative breast cancer. Mol Cancer. (2019) 18:42. doi: 10.1186/s12943-019-0988-0 30885232 PMC6421700

[36] Marquez-Ortiz RA Contreras-Zarate MJ Tesic V Alvarez-Eraso K Kwak G Littrell Z. IL13Ralpha2 promotes proliferation and outgrowth of breast cancer brain metastases. Clin Cancer Res. (2021) 27:6209–21. doi: 10.1158/1078-0432.CCR-21-0361 PMC8595859 34544797

[37] La Manna S Lee E Ouzounova M Di Natale C Novellino E Merlino A. Mimetics of suppressor of cytokine signaling 3: Novel potential therapeutics in triple breast cancer. Int J Cancer. (2018) 143:2177–86. doi: 10.1002/ijc.31594 29752723

[38] Du Z Lovly CM. Mechanisms of receptor tyrosine kinase activation in cancer. Mol Cancer. (2018) 17:58. doi: 10.1186/s12943-018-0782-4 29455648 PMC5817791

[39] Tebbutt N Pedersen MW Johns TG. Targeting the ERBB family in cancer: couples therapy. Nat Rev Cancer. (2013) 13:663–73. doi: 10.1038/nrc3559 23949426

[40] Wheeler DL Dunn EF Harari PM. Understanding resistance to EGFR inhibitors-impact on future treatment strategies. Nat Rev Clin Oncol. (2010) 7:493–507. doi: 10.1038/nrclinonc.2010.97 20551942 PMC2929287

[41] Wise R Zolkiewska A. Metalloprotease-dependent activation of EGFR modulates CD44(+)/CD24(-) populations in triple negative breast cancer cells through the MEK/ERK pathway. Breast Cancer Res Treat. (2017) 166:421–33. doi: 10.1007/s10549-017-4440-0 PMC5669811 28791489

[42] Bi J Wu Z Zhang X Zeng T Dai W Qiu N. TMEM25 inhibits monomeric EGFR-mediated STAT3 activation in basal state to suppress triple-negative breast cancer progression. Nat Commun. (2023) 14:2342. doi: 10.1038/s41467-023-38115-2 37095176 PMC10126118

[43] Tao JJ Castel P Radosevic-Robin N Elkabets M Auricchio N Aceto N. Antagonism of EGFR and HER3 enhances the response to inhibitors of the PI3K-Akt pathway in triple-negative breast cancer. Sci Signal. (2014) 7:ra29. doi: 10.1126/scisignal.2005125 24667376 PMC4283215

[44] Harrison PT Vyse S Huang PH. Rare epidermal growth factor receptor (EGFR) mutations in non-small cell lung cancer. Semin Cancer Biol. (2020) 61:167–79. doi: 10.1016/j.semcancer.2019.09.015 PMC7083237 31562956

[45] Jin H Shi Y Lv Y Yuan S Ramirez C Lieftink C. EGFR activation limits the response of liver cancer to lenvatinib. Nature. (2021) 595:730–34. doi: 10.1038/s41586-021-03741-7 34290403

[46] Prahallad A Sun C Huang S Di Nicolantonio F Salazar R Zecchin D. Unresponsiveness of colon cancer to BRAF(V600E) inhibition through feedback activation of EGFR. Nature. (2012) 483:100–03. doi: 10.1038/nature10868 22281684

[47] Nieto Y Nawaz F Jones RB Shpall EJ Nawaz S. Prognostic significance of overexpression and phosphorylation of epidermal growth factor receptor (EGFR) and the presence of truncated EGFRvIII in locoregionally advanced breast cancer. J Clin Oncol. (2007) 25:4405–13. doi: 10.1200/JCO.2006.09.8822 17906204

[48] Coley HM Shotton CF Ajose-Adeogun A Modjtahedi H Thomas H. Receptor tyrosine kinase (RTK) inhibition is effective in chemosensitising EGFR-expressing drug resistant human ovarian cancer cell lines when used in combination with cytotoxic agents. Biochem Pharmacol. (2006) 72:941–48. doi: 10.1016/j.bcp.2006.07.022 16934227

[49] Nielsen TO Hsu FD Jensen K Cheang M Karaca G Hu Z. Immunohistochemical and clinical characterization of the basal-like subtype of invasive breast carcinoma. Clin Cancer Res. (2004) 10:5367–74. doi: 10.1158/1078-0432.CCR-04-0220 15328174

[50] Rakha EA El-Sayed ME Green AR Lee AH Robertson JF Ellis IO. Prognostic markers in triple-negative breast cancer. Cancer. (2007) 109:25–32. doi: 10.1002/cncr.22381 17146782

[51] Liu H Paddock MN Wang H Murphy CJ Geck RC Navarro AJ. The INPP4B tumor suppressor modulates EGFR trafficking and promotes triple-negative breast cancer. Cancer Discovery. (2020) 10:1226–39. doi: 10.1158/2159-8290.CD-19-1262 PMC7415683 32513774

[52] Liu X Adorno-Cruz V Chang YF Jia Y Kawaguchi M Dashzeveg NK. EGFR inhibition blocks cancer stem cell clustering and lung metastasis of triple negative breast cancer. Theranostics. (2021) 11:6632–43. doi: 10.7150/thno.57706 PMC8120216 33995681

[53] Vaupel P. Hypoxia and aggressive tumor phenotype: implications for therapy and prognosis. Oncologist. (2008) 13 Suppl 3:21–6. doi: 10.1634/theoncologist.13-S3-21 18458121

[54] Shibuya M. Vascular endothelial growth factor (VEGF) and its receptor (VEGFR) signaling in angiogenesis: A crucial target for anti- and pro-angiogenic therapies. Genes Cancer. (2011) 2:1097–105. doi: 10.1177/1947601911423031 PMC3411125 22866201

[55] LeCouter J Moritz DR Li B Phillips GL Liang XH Gerber HP. Angiogenesis-independent endothelial protection of liver: role of VEGFR-1. Science. (2003) 299:890–93. doi: 10.1126/science.1079562 12574630

[56] Takahashi O Komaki R Smith PD Jurgensmeier JM Ryan A Bekele BN. Combined MEK and VEGFR inhibition in orthotopic human lung cancer models results in enhanced inhibition of tumor angiogenesis, growth, and metastasis. Clin Cancer Res. (2012) 18:1641–54. doi: 10.1158/1078-0432.CCR-11-2324 PMC3306446 22275507

[57] Jayasinghe C Simiantonaki N Habedank S Kirkpatrick CJ. The relevance of cell type- and tumor zone-specific VEGFR-2 activation in locally advanced colon cancer. J Exp Clin Cancer Res. (2015) 34:42. doi: 10.1186/s13046-015-0162-5 25967108 PMC4446839

[58] Sarkar S Rajput S Tripathi AK Mandal M. Targeted therapy against EGFR and VEGFR using ZD6474 enhances the therapeutic potential of UV-B phototherapy in breast cancer cells. Mol Cancer. (2013) 12:122. doi: 10.1186/1476-4598-12-122 24138843 PMC4015769

[59] Bai J Wu J Tang R Sun C Ji J Yin Z. Emodin, a natural anthraquinone, suppresses liver cancer in *vitro* and in *vivo* by regulating VEGFR(2) and miR-34a. Invest New Drugs. (2020) 38:229–45. doi: 10.1007/s10637-019-00777-5 30976957

[60] Spannuth WA Nick AM Jennings NB Armaiz-Pena GN Mangala LS Danes CG. Functional significance of VEGFR-2 on ovarian cancer cells. Int J Cancer. (2009) 124:1045–53. doi: 10.1002/ijc.24028 PMC2668132 19058181

[61] Bahhnassy A Mohanad M Shaarawy S Ismail MF El-Bastawisy A Ashmawy AM. Transforming growth factor-beta, insulin-like growth factor I/insulin-like growth factor I receptor and vascular endothelial growth factor-A: prognostic and predictive markers in triple-negative and non-triple-negative breast cancer. Mol Med Rep. (2015) 12:851–64. doi: 10.3892/mmr.2015.3560 PMC4438878 25824321

[62] Dong F Ruan S Wang J Xia Y Le K Xiao X. M2 macrophage-induced lncRNA PCAT6 facilitates tumorigenesis and angiogenesis of triple-negative breast cancer through modulation of VEGFR2. Cell Death Dis. (2020) 11:728. doi: 10.1038/s41419-020-02926-8 32908134 PMC7481779

[63] Ryden L Jirstrom K Haglund M Stal O Ferno M. Epidermal growth factor receptor and vascular endothelial growth factor receptor 2 are specific biomarkers in triple-negative breast cancer. Results from a controlled randomized trial with long-term follow-up. Breast Cancer Res Treat. (2010) 120:491–98. doi: 10.1007/s10549-010-0758-6 20135347

[64] Roberti MP Arriaga JM Bianchini M Quinta HR Bravo AI Levy EM. Protein expression changes during human triple negative breast cancer cell line progression to lymph node metastasis in a xenografted model in nude mice. Cancer Biol Ther. (2012) 13:1123–40. doi: 10.4161/cbt.21187 PMC3461818 22825326

[65] Eichhorn ME Strieth S Dellian M. Anti-vascular tumor therapy: recent advances, pitfalls and clinical perspectives. Drug Resist Update. (2004) 7:125–38. doi: 10.1016/j.drup.2004.03.001 15158768

[66] Noiseux N Boucher CH Cartier R Sirois MG. Bolus endovascular PDGFR-beta antisense treatment suppressed intimal hyperplasia in a rat carotid injury model. Circulation. (2000) 102:1330–36. doi: 10.1161/01.cir.102.11.1330 10982551

[67] Cristofanilli M Morandi P Krishnamurthy S Reuben JM Lee BN Francis D. Imatinib mesylate (Gleevec) in advanced breast cancer-expressing C-Kit or PDGFR-beta: clinical activity and biological correlations. Ann Oncol. (2008) 19:1713–19. doi: 10.1093/annonc/mdn352 PMC2735063 18515258

[68] Abramsson A Lindblom P Betsholtz C. Endothelial and nonendothelial sources of PDGF-B regulate pericyte recruitment and influence vascular pattern formation in tumors. J Clin Invest. (2003) 112:1142–51. doi: 10.1172/JCI18549 PMC213487 14561699

[69] Riess JW Neal JW. Targeting FGFR, ephrins, Mer, MET, and PDGFR-alpha in non-small cell lung cancer. J Thorac Oncol. (2011) 6:S1797–98. doi: 10.1097/01.JTO.0000407562.07029.52 22005534

[70] Kuwai T Nakamura T Sasaki T Kitadai Y Kim JS Langley RR. Targeting the EGFR, VEGFR, and PDGFR on colon cancer cells and stromal cells is required for therapy. Clin Exp Metastasis. (2008) 25:477–89. doi: 10.1007/s10585-008-9153-7 18324358

[71] Primac I Maquoi E Blacher S Heljasvaara R Van Deun J Smeland HY. Stromal integrin alpha11 regulates PDGFR-beta signaling and promotes breast cancer progression. J Clin Invest. (2019) 129:4609–28. doi: 10.1172/JCI125890 PMC6819106 31287804

[72] Werner TL Wade ML Agarwal N Boucher K Patel J Luebke A. A pilot study of JI-101, an inhibitor of VEGFR-2, PDGFR-beta, and EphB4 receptors, in combination with everolimus and as a single agent in an ovarian cancer expansion cohort. Invest New Drugs. (2015) 33:1217–24. doi: 10.1007/s10637-015-0288-5 26365907

[73] Hollier BG Tinnirello AA Werden SJ Evans KW Taube JH Sarkar TR. FOXC2 expression links epithelial-mesenchymal transition and stem cell properties in breast cancer. Cancer Res. (2013) 73:1981–92. doi: 10.1158/0008-5472.CAN-12-2962 PMC3602160 23378344

[74] Itoh N Ornitz DM. Functional evolutionary history of the mouse Fgf gene family. Dev Dyn. (2008) 237:18–27. doi: 10.1002/dvdy.21388 18058912

[75] Wu YM Su F Kalyana-Sundaram S Khazanov N Ateeq B Cao X. Identification of targetable FGFR gene fusions in diverse cancers. Cancer Discovery. (2013) 3:636–47. doi: 10.1158/2159-8290.CD-13-0050 PMC3694764 23558953

[76] Lin L Chamberlain L Pak ML Nagarajan A Gupta R Zhu LJ. A large-scale RNAi-based mouse tumorigenesis screen identifies new lung cancer tumor suppressors that repress FGFR signaling. Cancer Discovery. (2014) 4:1168–81. doi: 10.1158/2159-8290.CD-13-0747 PMC4184919 25015643

[77] Andre F Bachelot T Campone M Dalenc F Perez-Garcia JM Hurvitz SA. Targeting FGFR with dovitinib (TKI258): preclinical and clinical data in breast cancer. Clin Cancer Res. (2013) 19:3693–702. doi: 10.1158/1078-0432.CCR-13-0190 23658459

[78] Zecchini S Bombardelli L Decio A Bianchi M Mazzarol G Sanguineti F. The adhesion molecule NCAM promotes ovarian cancer progression via FGFR signalling. EMBO Mol Med. (2011) 3:480–94. doi: 10.1002/emmm.201100152 PMC3377089 21739604

[79] Ye F Dewanjee S Li Y Jha NK Chen ZS Kumar A. Advancements in clinical aspects of targeted therapy and immunotherapy in breast cancer. Mol Cancer. (2023) 22:105. doi: 10.1186/s12943-023-01805-y 37415164 PMC10324146

[80] Helsten T Elkin S Arthur E Tomson BN Carter J Kurzrock R. The FGFR landscape in cancer: analysis of 4,853 tumors by next-generation sequencing. Clin Cancer Res. (2016) 22:259–67. doi: 10.1158/1078-0432.CCR-14-3212 26373574

[81] Jung SY Yi JY Kim MH Song KH Kang SM Ahn J. IM-412 inhibits the invasion of human breast carcinoma cells by blocking FGFR-mediated signaling. Oncol Rep. (2015) 34:2731–37. doi: 10.3892/or.2015.4249 26351897

[82] Fillmore CM Gupta PB Rudnick JA Caballero S Keller PJ Lander ES. Estrogen expands breast cancer stem-like cells through paracrine FGF/Tbx3 signaling. Proc Natl Acad Sci U.S.A. (2010) 107:21737–42. doi: 10.1073/pnas.1007863107 PMC3003123 21098263

[83] Brooks AJ Dai W O’Mara ML Abankwa D Chhabra Y Pelekanos RA. Mechanism of activation of protein kinase JAK2 by the growth hormone receptor. Science. (2014) 344:1249783. doi: 10.1126/science.1249783 24833397

[84] Lacronique V Boureux A Valle VD Poirel H Quang CT Mauchauffe M. A TEL-JAK2 fusion protein with constitutive kinase activity in human leukemia. Science. (1997) 278:1309–12. doi: 10.1126/science.278.5341.1309 9360930

[85] Nagai H Kim YS Lee KT Chu MY Konishi N Fujimoto J. Inactivation of SSI-1, a JAK/STAT inhibitor, in human hepatocellular carcinomas, as revealed by two-dimensional electrophoresis. J Hepatol. (2001) 34:416–21. doi: 10.1016/s0168-8278(00)00038-6 11322203

[86] Huang L Ma B Ma J Wang F. Fractalkine/CX3CR1 axis modulated the development of pancreatic ductal adenocarcinoma via JAK/STAT signaling pathway. Biochem Biophys Res Commun. (2017) 493:1510–17. doi: 10.1016/j.bbrc.2017.10.006 28986258

[87] Balko JM Schwarz LJ Luo N Estrada MV Giltnane JM Davila-Gonzalez D. Triple-negative breast cancers with amplification of JAK2 at the 9p24 locus demonstrate JAK2-specific dependence. Sci Transl Med. (2016) 8:334ra53. doi: 10.1126/scitranslmed.aad3001 PMC5256931 27075627

[88] Bromberg J. Stat proteins and oncogenesis. J Clin Invest. (2002) 109:1139–42. doi: 10.1172/JCI15617 PMC150969 11994401

[89] Cimica V Chen HC Iyer JK Reich NC. Dynamics of the STAT3 transcription factor: nuclear import dependent on Ran and importin-beta1. PloS One. (2011) 6:e20188. doi: 10.1371/journal.pone.0020188 21625522 PMC3098288

[90] Wu S Lu J Zhu H Wu F Mo Y Xie L. A novel axis of circKIF4A-miR-637-STAT3 promotes brain metastasis in triple-negative breast cancer. Cancer Lett. (2024) 581:216508. doi: 10.1016/j.canlet.2023.216508 38029538

[91] Aaronson DS Horvath CM. A road map for those who don’t know JAK-STAT. Science. (2002) 296:1653–55. doi: 10.1126/science.1071545 12040185

[92] Darnell JJ Kerr IM Stark GR. Jak-STAT pathways and transcriptional activation in response to IFNs and other extracellular signaling proteins. Science. (1994) 264:1415–21. doi: 10.1126/science.8197455 8197455

[93] Yates LR Knappskog S Wedge D Farmery J Gonzalez S Martincorena I. Genomic evolution of breast cancer metastasis and relapse. Cancer Cell. (2017) 32:169–84. doi: 10.1016/j.ccell.2017.07.005 PMC5559645 28810143

[94] Park SY Lee CJ Choi JH Kim JH Kim JW Kim JY. The JAK2/STAT3/CCND2 Axis promotes colorectal Cancer stem cell persistence and radioresistance. J Exp Clin Cancer Res. (2019) 38:399. doi: 10.1186/s13046-019-1405-7 31511084 PMC6737692

[95] Choi DS Blanco E Kim YS Rodriguez AA Zhao H Huang TH. Chloroquine eliminates cancer stem cells through deregulation of Jak2 and DNMT1. Stem Cells. (2014) 32:2309–23. doi: 10.1002/stem.1746 PMC4138251 24809620

[96] Zhao C Chen HY Zhao F Feng HJ Su JP. Acylglycerol kinase promotes paclitaxel resistance in nasopharyngeal carcinoma cells by regulating FOXM1 via the JAK2/STAT3 pathway. Cytokine. (2021) 148:155595. doi: 10.1016/j.cyto.2021.155595 34116927

[97] Xu Y Zhang J Wu J Zhong S Li H. Inhibition of JAK2 reverses paclitaxel resistance in human ovarian cancer cells. Int J Gynecol Cancer. (2015) 25:1557–64. doi: 10.1097/IGC.0000000000000550 26360705

[98] Zhang J Zhao J Zhang W Liu G Yin D Li J. Establishment of paclitaxel-resistant cell line and the underlying mechanism on drug resistance. Int J Gynecol Cancer. (2012) 22:1450–56. doi: 10.1097/IGC.0b013e31826e2382 23051955

[99] Cheon JH Kim KS Yadav DK Kim M Kim HS Yoon S. The JAK2 inhibitors CEP-33779 and NVP-BSK805 have high P-gp inhibitory activity and sensitize drug-resistant cancer cells to vincristine. Biochem Biophys Res Commun. (2017) 490:1176–82. doi: 10.1016/j.bbrc.2017.06.178 28669723

[100] Tanei T Choi DS Rodriguez AA Liang DH Dobrolecki L Ghosh M. Antitumor activity of Cetuximab in combination with Ixabepilone on triple negative breast cancer stem cells. Breast Cancer Res. (2016) 18:6. doi: 10.1186/s13058-015-0662-4 26757880 PMC4711100

[101] Carey LA Rugo HS Marcom PK Mayer EL Esteva FJ Ma CX. TBCRC 001: randomized phase II study of cetuximab in combination with carboplatin in stage IV triple-negative breast cancer. J Clin Oncol. (2012) 30:2615–23. doi: 10.1200/JCO.2010.34.5579 PMC3413275 22665533

[102] Xie W Zhao H Wang F Wang Y He Y Wang T. A novel humanized Frizzled-7-targeting antibody enhances antitumor effects of Bevacizumab against triple-negative breast cancer via blocking Wnt/beta-catenin signaling pathway. J Exp Clin Cancer Res. (2021) 40:30. doi: 10.1186/s13046-020-01800-x 33436039 PMC7802198

[103] Alraouji NN Al-Mohanna FH Ghebeh H Arafah M Almeer R Al-Tweigeri T. Tocilizumab potentiates cisplatin cytotoxicity and targets cancer stem cells in triple-negative breast cancer. Mol Carcinog. (2020) 59:1041–51. doi: 10.1002/mc.23234 32537818

[104] Yang C Zhang J Zhang Y Ji F Chen Y Zhu T. Low-dose apatinib combined with neoadjuvant chemotherapy in the treatment of early-stage triple-negative breast cancer (LANCET): a single-center, single-arm, phase II trial. Ther Adv Med Oncol. (2022) 14:7437211. doi: 10.1177/17588359221118053 PMC9379563 35983024

[105] Liu J Liu Q Li Y Li Q Su F Yao H. Efficacy and safety of camrelizumab combined with apatinib in advanced triple-negative breast cancer: an open-label phase II trial. J Immunother Cancer. (2020) 8:e000696. doi: 10.1136/jitc-2020-000696 32448804 PMC7252975

[106] Liu J Wang Y Tian Z Lin Y Li H Zhu Z. Multicenter phase II trial of Camrelizumab combined with Apatinib and Eribulin in heavily pretreated patients with advanced triple-negative breast cancer. Nat Commun. (2022) 13:3011. doi: 10.1038/s41467-022-30569-0 35641481 PMC9156739

[107] Wang J Sun T Ouyang Q Han Y Xu B. A phase Ib study of TQB2450 plus anlotinib in patients with advanced triple-negative breast cancer. Iscience. (2023) 26:106876. doi: 10.1016/j.isci.2023.106876 37275528 PMC10238930

[108] Huang JY Xie XF Chen XL Zhang QY Chen LP Bai X. A single-arm phase II clinical trial of anlotinib combined with chemotherapy for the treatment of metastatic triple-negative breast cancer. Front Oncol. (2023) 13:1122294. doi: 10.3389/fonc.2023.1122294 37124484 PMC10130368

[109] Bernsdorf M Ingvar C Jorgensen L Tuxen MK Jakobsen EH Saetersdal A. Effect of adding gefitinib to neoadjuvant chemotherapy in estrogen receptor negative early breast cancer in a randomized phase II trial. Breast Cancer Res Treat. (2011) 126:463–70. doi: 10.1007/s10549-011-1352-2 21234672

[110] Symonds L Linden H Gadi V Korde L Rodler E Gralow J. Combined targeted therapies for first-line treatment of metastatic triple negative breast cancer-A phase II trial of weekly nab-paclitaxel and bevacizumab followed by maintenance targeted therapy with bevacizumab and erlotinib. Clin Breast Cancer. (2019) 19:e283–96. doi: 10.1016/j.clbc.2018.12.008 PMC6440867 30737173

[111] Stover DG Gil DAC Brock J Guo H Overmoyer B Balko J. Phase II study of ruxolitinib, a selective JAK1/2 inhibitor, in patients with metastatic triple-negative breast cancer. NPJ Breast Cancer. (2018) 4:10. doi: 10.1038/s41523-018-0060-z 29761158 PMC5935675

[112] O’Shaughnessy J DeMichele A Ma CX Richards P Yardley DA Wright GS. A randomized, double-blind, phase 2 study of ruxolitinib or placebo in combination with capecitabine in patients with advanced HER2-negative breast cancer and elevated C-reactive protein, a marker of systemic inflammation. Breast Cancer Res Treat. (2018) 170:547–57. doi: 10.1007/s10549-018-4770-6 29675680

[113] Lynce F Williams JT Regan MM Bunnell CA Freedman RA Tolaney SM. Phase I study of JAK1/2 inhibitor ruxolitinib with weekly paclitaxel for the treatment of HER2-negative metastatic breast cancer. Cancer Chemother Pharmacol. (2021) 87:673–79. doi: 10.1007/s00280-021-04245-x 33585999

[114] Kang DY Sp N Kim DH Joung YH Lee HG Park YM. Salidroside inhibits migration, invasion and angiogenesis of MDA−MB 231 TNBC cells by regulating EGFR/Jak2/STAT3 signaling via MMP2. Int J Oncol. (2018) 53:877–85. doi: 10.3892/ijo.2018.4430 29901185

[115] Kim S Park JM Park S Jung E Ko D Park M. Suppression of TNBC metastasis by doxazosin, a novel dual inhibitor of c-MET/EGFR. J Exp Clin Cancer Res. (2023) 42:292. doi: 10.1186/s13046-023-02866-z 37924112 PMC10625208

[116] Li YC Wong CN Hsu FT Chen JH Yang CC Liu HH. Accessing apoptosis induction and metastasis inhibition effect of magnolol on triple negative breast cancer *in vitro* . In Vivo. (2023) 37:1028–36. doi: 10.21873/invivo.13177 PMC10188043 37103080

[117] Sebastian A Pandey V Mohan CD Chia YT Rangappa S Mathai J. Novel adamantanyl-based thiadiazolyl pyrazoles targeting EGFR in triple-negative breast cancer. ACS Omega. (2016) 1:1412–24. doi: 10.1021/acsomega.6b00251 PMC6044684 30023509

[118] Shieh JM Chen YC Lin YC Lin JN Chen WC Chen YY. Demethoxycurcumin inhibits energy metabolic and oncogenic signaling pathways through AMPK activation in triple-negative breast cancer cells. J Agric Food Chem. (2013) 61:6366–75. doi: 10.1021/jf4012455 23777448

[119] Maharjan S Lee MG Kim SY Lee KS Nam KS. Morin sensitizes MDA-MB-231 triple-negative breast cancer cells to doxorubicin cytotoxicity by suppressing FOXM1 and attenuating EGFR/STAT3 signaling pathways. Pharm (Basel). (2023) 16:672. doi: 10.3390/ph16050672 PMC10222377 37242455

[120] Garay JP Karakas B Abukhdeir AM Cosgrove DP Gustin JP Higgins MJ. The growth response to androgen receptor signaling in ERalpha-negative human breast cells is dependent on p21 and mediated by MAPK activation. Breast Cancer Res. (2012) 14:R27. doi: 10.1186/bcr3112 22321971 PMC3496145

[121] Kim JH Choi HS Lee DS. Primaquine inhibits the endosomal trafficking and nuclear localization of EGFR and induces the apoptosis of breast cancer cells by nuclear EGFR/stat3-mediated c-myc downregulation. Int J Mol Sci. (2021) 22:12961. doi: 10.3390/ijms222312961 34884765 PMC8657416

[122] Lee MM Chan BD Wong WY Qu Z Chan MS Leung TW. Anti-cancer Activity of Centipeda minima Extract in Triple Negative Breast Cancer via Inhibition of AKT, NF-kappaB, and STAT3 Signaling Pathways. Front Oncol. (2020) 10:491. doi: 10.3389/fonc.2020.00491 32328465 PMC7160338

[123] Huang Q Li S Zhang L Qiao X Zhang Y Zhao X. CAPE-pNO(2) inhibited the growth and metastasis of triple-negative breast cancer via the EGFR/STAT3/akt/E-cadherin signaling pathway. Front Oncol. (2019) 9:461. doi: 10.3389/fonc.2019.00461 31214503 PMC6558049

[124] Huang L Wong CC Mackenzie GG Sun Y Cheng KW Vrankova K. Phospho-aspirin (MDC-22) inhibits breast cancer in preclinical animal models: an effect mediated by EGFR inhibition, p53 acetylation and oxidative stress. BMC Cancer. (2014) 14:141. doi: 10.1186/1471-2407-14-141 24575839 PMC3941604

[125] Mehta R Katta H Alimirah F Patel R Murillo G Peng X. Deguelin action involves c-Met and EGFR signaling pathways in triple negative breast cancer cells. PloS One. (2013) 8:e65113. doi: 10.1371/journal.pone.0065113 23762292 PMC3677900

[126] Suh YA Kim JH Sung MA Boo HJ Yun HJ Lee SH. A novel antitumor activity of deguelin targeting the insulin-like growth factor (IGF) receptor pathway via up-regulation of IGF-binding protein-3 expression in breast cancer. Cancer Lett. (2013) 332:102–09. doi: 10.1016/j.canlet.2013.01.022 PMC3638119 23348700

[127] Yamashita N Kondo M Zhao S Li W Koike K Nemoto K. Picrasidine G decreases viability of MDA-MB 468 EGFR-overexpressing triple-negative breast cancer cells through inhibition of EGFR/STAT3 signaling pathway. Bioorg Med Chem Lett. (2017) 27:2608–12. doi: 10.1016/j.bmcl.2017.03.061 28427809

[128] Su JC Mar AC Wu SH Tai WT Chu PY Wu CY. Disrupting VEGF-A paracrine and autocrine loops by targeting SHP-1 suppresses triple negative breast cancer metastasis. Sci Rep. (2016) 6:28888. doi: 10.1038/srep28888 27364975 PMC4929457

[129] Tian J Chen X Fu S Zhang R Pan L Cao Y. Bazedoxifene is a novel IL-6/GP130 inhibitor for treating triple-negative breast cancer. Breast Cancer Res Treat. (2019) 175:553–66. doi: 10.1007/s10549-019-05183-2 30852762

[130] Li H Xiao H Lin L Jou D Kumari V Lin J. Drug design targeting protein-protein interactions (PPIs) using multiple ligand simultaneous docking (MLSD) and drug repositioning: discovery of raloxifene and bazedoxifene as novel inhibitors of IL-6/GP130 interface. J Med Chem. (2014) 57:632–41. doi: 10.1021/jm401144z 24456369

[131] Shi W Yan D Zhao C Xiao M Wang Y Ma H. Inhibition of IL-6/STAT3 signaling in human cancer cells using Evista. Biochem Biophys Res Commun. (2017) 491:159–65. doi: 10.1016/j.bbrc.2017.07.067 28711499

[132] Viswanadhapalli S Luo Y Sareddy GR Santhamma B Zhou M Li M. EC359: A first-in-class small-molecule inhibitor for targeting oncogenic LIFR signaling in triple-negative breast cancer. Mol Cancer Ther. (2019) 18:1341–54. doi: 10.1158/1535-7163.MCT-18-1258 PMC6677593 31142661

[133] Yang J Qian S Cai X Lu W Hu C Sun X. Chikusetsusaponin IVa butyl ester (CS-IVa-be), a novel IL6R antagonist, inhibits IL6/STAT3 signaling pathway and induces cancer cell apoptosis. Mol Cancer Ther. (2016) 15:1190–200. doi: 10.1158/1535-7163.MCT-15-0551 26929249

[134] Bouaouiche S Ghione S Sghaier R Burgy O Racoeur C Derangere V. Nitric oxide-releasing drug glyceryl trinitrate targets JAK2/STAT3 signaling, migration and invasion of triple-negative breast cancer cells. Int J Mol Sci. (2021) 22:8449. doi: 10.3390/ijms22168449 34445170 PMC8395103

[135] Lee J Hahm ER Singh SV. Withaferin A inhibits activation of signal transducer and activator of transcription 3 in human breast cancer cells. Carcinogenesis. (2010) 31:1991–98. doi: 10.1093/carcin/bgq175 PMC2966554 20724373

[136] Lin KL Su JC Chien CM Tseng CH Chen YL Chang LS. Naphtho[1,2-b]furan-4,5-dione disrupts Janus kinase-2 and induces apoptosis in breast cancer MDA-MB-231 cells. Toxicol Vitro. (2010) 24:1158–67. doi: 10.1016/j.tiv.2010.02.019 20197088

[137] Yang Y Zhou H Liu W Wu J Yue X Wang J. Ganoderic acid A exerts antitumor activity against MDA-MB-231 human breast cancer cells by inhibiting the Janus kinase 2/signal transducer and activator of transcription 3 signaling pathway. Oncol Lett. (2018) 16:6515–21. doi: 10.3892/ol.2018.9475 PMC6202552 30405790

[138] Qiu C Zhang T Zhu X Qiu J Jiang K Zhao G. Methylseleninic acid suppresses breast cancer growth via the JAK2/STAT3 pathway. Reprod Sci. (2019) 26:829–38. doi: 10.1177/1933719118815582 30526368

[139] Kalimutho M Sinha D Mittal D Srihari S Nanayakkara D Shafique S. Blockade of PDGFRbeta circumvents resistance to MEK-JAK inhibition via intratumoral CD8(+) T-cells infiltration in triple-negative breast cancer. J Exp Clin Cancer Res. (2019) 38:85. doi: 10.1186/s13046-019-1075-5 30777101 PMC6379987

[140] Khanna P Lee JS Sereemaspun A Lee H Baeg GH. GRAMD1B regulates cell migration in breast cancer cells through JAK/STAT and Akt signaling. Sci Rep. (2018) 8:9511. doi: 10.1038/s41598-018-27864-6 29934528 PMC6015000

[141] Jeon M You D Bae SY Kim SW Nam SJ Kim HH. Dimerization of EGFR and HER2 induces breast cancer cell motility through STAT1-dependent ACTA2 induction. Oncotarget. (2017) 8:50570–81. doi: 10.18632/oncotarget.10843 PMC5584169 28881584

[142] Shan H Yao S Ye Y Yu Q. 3-Deoxy-2beta,16-dihydroxynagilactone E, a natural compound from Podocarpus nagi, preferentially inhibits JAK2/STAT3 signaling by allosterically interacting with the regulatory domain of JAK2 and induces apoptosis of cancer cells. Acta Pharmacol Sin. (2019) 40:1578–86. doi: 10.1038/s41401-019-0254-4 PMC7471446 31201357

[143] Jang H Ko H Song K Kim YS. A sesquiterpenoid from farfarae flos induces apoptosis of MDA-MB-231 human breast cancer cells through inhibition of JAK-STAT3 signaling. Biomolecules. (2019) 9:278. doi: 10.3390/biom9070278 31337063 PMC6681226

[144] Byun HJ Darvin P Kang DY Sp N Joung YH Park JH. Silibinin downregulates MMP2 expression via Jak2/STAT3 pathway and inhibits the migration and invasive potential in MDA-MB-231 cells. Oncol Rep. (2017) 37:3270–78. doi: 10.3892/or.2017.5588 28440514

[145] Binienda A Ziolkowska S Pluciennik E. The anticancer properties of silibinin: its molecular mechanism and therapeutic effect in breast cancer. Anticancer Agents Med Chem. (2020) 20:1787–96. doi: 10.2174/1871520620666191220142741 31858905

[146] Chen D Ma Y Li P Liu M Fang Y Zhang J. Piperlongumine induces apoptosis and synergizes with doxorubicin by inhibiting the JAK2-STAT3 pathway in triple-negative breast cancer. Molecules. (2019) 24:2338. doi: 10.3390/molecules24122338 31242627 PMC6631638

[147] Shakya R Park GH Joo SH Shim JH Choi JS. Hydroxyzine induces cell death in triple-negative breast cancer cells via mitochondrial superoxide and modulation of jak2/STAT3 signaling. Biomol Ther (Seoul). (2022) 30:585–92. doi: 10.4062/biomolther.2022.121 PMC9622314 36305293

[148] Schust J Sperl B Hollis A Mayer TU Berg T. Stattic: a small-molecule inhibitor of STAT3 activation and dimerization. Chem Biol. (2006) 13:1235–42. doi: 10.1016/j.chembiol.2006.09.018 17114005

[149] Poria DK Sheshadri N Balamurugan K Sharan S Sterneck E. The STAT3 inhibitor Stattic acts independently of STAT3 to decrease histone acetylation and modulate gene expression. J Biol Chem. (2021) 296:100220. doi: 10.1074/jbc.RA120.016645 33839684 PMC7948742

[150] Song H Wang R Wang S Lin J. A low-molecular-weight compound discovered through virtual database screening inhibits Stat3 function in breast cancer cells. Proc Natl Acad Sci U.S.A. (2005) 102:4700–05. doi: 10.1073/pnas.0409894102 PMC555708 15781862

[151] Lin L Hutzen B Zuo M Ball S Deangelis S Foust E. Novel STAT3 phosphorylation inhibitors exhibit potent growth-suppressive activity in pancreatic and breast cancer cells. Cancer Res. (2010) 70:2445–54. doi: 10.1158/0008-5472.CAN-09-2468 PMC2843552 20215512

[152] Zinzalla G Haque MR Basu BP Anderson J Kaye SL Haider S. A novel small-molecule inhibitor of IL-6 signalling. Bioorg Med Chem Lett. (2010) 20:7029–32. doi: 10.1016/j.bmcl.2010.09.117 21030257

[153] Lin L Hutzen B Li PK Ball S Zuo M DeAngelis S. A novel small molecule, LLL12, inhibits STAT3 phosphorylation and activities and exhibits potent growth-suppressive activity in human cancer cells. Neoplasia. (2010) 12:39–50. doi: 10.1593/neo.91196 20072652 PMC2805882

[154] Noori S Rezaei TM Deravi N Mahboobi RM Zarghi A. Naringenin enhances the anti-cancer effect of cyclophosphamide against MDA-MB-231 breast cancer cells via targeting the STAT3 signaling pathway. Iran J Pharm Res. (2020) 19:122–33. doi: 10.22037/ijpr.2020.113103.14112 PMC7757999 33680016

[155] Siddiquee K Zhang S Guida WC Blaskovich MA Greedy B Lawrence HR. Selective chemical probe inhibitor of Stat3, identified through structure-based virtual screening, induces antitumor activity. Proc Natl Acad Sci U.S.A. (2007) 104:7391–96. doi: 10.1073/pnas.0609757104 PMC1863497 17463090

[156] Locken H Clamor C Muller K. Napabucasin and related heterocycle-fused naphthoquinones as STAT3 inhibitors with antiproliferative activity against cancer cells. J Nat Prod. (2018) 81:1636–44. doi: 10.1021/acs.jnatprod.8b00247 30003778

[157] Cai G Yu W Song D Zhang W Guo J Zhu J. Discovery of fluorescent coumarin-benzothiophene 1, 1-dioxide conjugates as mitochondria-targeting antitumor STAT3 inhibitors. Eur J Med Chem. (2019) 174:236–51. doi: 10.1016/j.ejmech.2019.04.024 31048139

[158] Park SK Byun WS Lee S Han YT Jeong YS Jang K. A novel small molecule STAT3 inhibitor SLSI-1216 suppresses proliferation and tumor growth of triple-negative breast cancer cells through apoptotic induction. Biochem Pharmacol. (2020) 178:114053. doi: 10.1016/j.bcp.2020.114053 32450253

[159] Yue P Zhu Y Brotherton-Pleiss C Fu W Verma N Chen J. Novel potent azetidine-based compounds irreversibly inhibit Stat3 activation and induce antitumor response against human breast tumor growth in *vivo* . Cancer Lett. (2022) 534:215613. doi: 10.1016/j.canlet.2022.215613 35276290 PMC9867837

[160] Yang Z Xu H Yang Y Duan C Zhang P Wang Y. Synthesis and evaluation of naphthalene derivatives as potent STAT3 inhibitors and agents against triple-negative breast cancer growth and metastasis. Breast Cancer Res Treat. (2023) 197:255–67. doi: 10.1007/s10549-022-06790-2 36369502

[161] Byun WS Lim H Hong J Bae ES Lee SB Kim Y. Design, synthesis, and biological activity of marinacarboline analogues as STAT3 pathway inhibitors for docetaxel-resistant triple-negative breast cancer. J Med Chem. (2023) 66:3106–33. doi: 10.1021/acs.jmedchem.2c01115 36786551

[162] Xie Y Zhu S Chen L Liu H Peng T Ming Z. An isoxazoloquinone derivative inhibits tumor growth by targeting STAT3 and triggering its ubiquitin-dependent degradation. Cancers (Basel). (2023) 15:2424. doi: 10.3390/cancers15092424 37173892 PMC10177496

[163] Qi Y Wu H Zhu T Liu Z Liu C Yan C. Acetyl-cinobufagin suppresses triple-negative breast cancer progression by inhibiting the STAT3 pathway. Aging (Albany Ny). (2023) 15:8258–74. doi: 10.18632/aging.204967 PMC10497018 37651362

[164] Feng T Cao W Shen W Zhang L Gu X Guo Y. Arctigenin inhibits STAT3 and exhibits anticancer potential in human triple-negative breast cancer therapy. Oncotarget. (2017) 8:329–44. doi: 10.18632/oncotarget.13393 PMC5352123 27861147

[165] Zhang W Yu W Cai G Zhu J Zhang C Li S. A new synthetic derivative of cryptotanshinone KYZ3 as STAT3 inhibitor for triple-negative breast cancer therapy. Cell Death Dis. (2018) 9:1098. doi: 10.1038/s41419-018-1139-z 30368518 PMC6204138

[166] Kim SL Choi HS Kim JH Jeong DK Kim KS Lee DS. Dihydrotanshinone-induced NOX5 activation inhibits breast cancer stem cell through the ROS/stat3 signaling pathway. Oxid Med Cell Longev. (2019) 2019:9296439. doi: 10.1155/2019/9296439 31019654 PMC6451810

[167] He J Wei X Li S Quan X Li R Du H. DT-13 suppresses breast cancer metastasis by modulating PLOD2 in the adipocytes microenvironment. Phytomedicine. (2019) 59:152778. doi: 10.1016/j.phymed.2018.12.001 31005809

[168] Lan T Wang L Xu Q Liu W Jin H Mao W. Growth inhibitory effect of Cucurbitacin E on breast cancer cells. Int J Clin Exp Pathol. (2013) 6:1799–805. PMC3759486 24040444

[169] Nguyen T Pham T Nguyen C Truong TN Bishop C Doan N. Phytochemistry and cytotoxic activity of aquilaria crassna pericarp on MDA-MB-468 cell lines. ACS Omega. (2023) 8:42356–66. doi: 10.1021/acsomega.3c04656 PMC10652264 38024711

[170] Kong Y Chen J Zhou Z Xia H Qiu MH Chen C. Cucurbitacin E induces cell cycle G2/M phase arrest and apoptosis in triple negative breast cancer. PloS One. (2014) 9:e103760. doi: 10.1371/journal.pone.0103760 25072848 PMC4114842

[171] Gyamfi J Lee YH Min BS Choi J. Niclosamide reverses adipocyte induced epithelial-mesenchymal transition in breast cancer cells via suppression of the interleukin-6/STAT3 signalling axis. Sci Rep. (2019) 9:11336. doi: 10.1038/s41598-019-47707-2 31383893 PMC6683291

[172] Ss PS Krishnamurthy PT Ghanta VR Chintamaneni PK. Phenyl boronic acid-modified lipid nanocarriers of niclosamide for targeting triple-negative breast cancer. Nanomedicine (Lond). (2020) 15:1551–65. doi: 10.2217/nnm-2020-0003 32618501

[173] Pindiprolu S Chintamaneni PK Krishnamurthy PT Ratna SGK. Formulation-optimization of solid lipid nanocarrier system of STAT3 inhibitor to improve its activity in triple negative breast cancer cells. Drug Dev Ind Pharm. (2019) 45:304–13. doi: 10.1080/03639045.2018.1539496 30348020

[174] Ko H Lee JH Kim HS Kim T Han YT Suh YG. Novel galiellalactone analogues can target STAT3 phosphorylation and cause apoptosis in triple-negative breast cancer. Biomolecules. (2019) 9:170. doi: 10.3390/biom9050170 31058868 PMC6571922

[175] Yang F Hu M Lei Q Xia Y Zhu Y Song X. Nifuroxazide induces apoptosis and impairs pulmonary metastasis in breast cancer model. Cell Death Dis. (2015) 6:e1701. doi: 10.1038/cddis.2015.63 25811798 PMC4385941

[176] Wang X Shi W Wang X Lu JJ He P Zhang H. Nifuroxazide boosts the anticancer efficacy of palbociclib-induced senescence by dual inhibition of STAT3 and CDK2 in triple-negative breast cancer. Cell Death Discovery. (2023) 9:355. doi: 10.1038/s41420-023-01658-w 37752122 PMC10522654

[177] Pan L Chen X Fu S Yu W Li C Wang T. LLY17, a novel small molecule STAT3 inhibitor induces apoptosis and suppresses cell migration and tumor growth in triple-negative breast cancer. Breast Cancer Res Treat. (2020) 181:31–41. doi: 10.1007/s10549-020-05613-6 32240456

[178] Liu Z Ge X Gu Y Huang Y Liu H Yu M. Small molecule STAT3 inhibitor, 6Br-6a suppresses breast cancer growth in *vitro* and in *vivo* . BioMed Pharmacother. (2020) 121:109502. doi: 10.1016/j.biopha.2019.109502 31707351

[179] Khan MW Saadalla A Ewida AH Al-Katranji K Al-Saoudi G Giaccone ZT. The STAT3 inhibitor pyrimethamine displays anti-cancer and immune stimulatory effects in murine models of breast cancer. Cancer Immunol Immunother. (2018) 67:13–23. doi: 10.1007/s00262-017-2057-0 28875329 PMC5783191

[180] Brown JI Wang P Wong A Petrova B Persaud R Soukhtehzari S. Cycloguanil and analogues potently target DHFR in cancer cells to elicit anti-cancer activity. Metabolites. (2023) 13:151. doi: 10.3390/metabo13020151 36837770 PMC9961069

[181] Li Y Gan C Zhang Y Yu Y Fan C Deng Y. Inhibition of stat3 signaling pathway by natural product pectolinarigenin attenuates breast cancer metastasis. Front Pharmacol. (2019) 10:1195. doi: 10.3389/fphar.2019.01195 31649548 PMC6796319

[182] Oh E Kim YJ An H Sung D Cho TM Farrand L. Flubendazole elicits anti-metastatic effects in triple-negative breast cancer via STAT3 inhibition. Int J Cancer. (2018) 143:1978–93. doi: 10.1002/ijc.31585 29744876

[183] Dinakar YH Kumar H Mudavath SL Jain R Ajmeer R Jain V. Role of STAT3 in the initiation, progression, proliferation and metastasis of breast cancer and strategies to deliver JAK and STAT3 inhibitors. Life Sci. (2022) 309:120996. doi: 10.1016/j.lfs.2022.120996 36170890

[184] Yang B Shen JW Zhou DH Zhao YP Wang WQ Zhu Y. Precise discovery of a STAT3 inhibitor from Eupatorium lindleyanum and evaluation of its activity of anti-triple-negative breast cancer. Nat Prod Res. (2019) 33:477–85. doi: 10.1080/14786419.2017.1396596 29086600

[185] Lou C Chen Y Zhang J Yang B Zhao H. Eupalinolide J suppresses the growth of triple-negative breast cancer cells via targeting STAT3 signaling pathway. Front Pharmacol. (2019) 10:1071. doi: 10.3389/fphar.2019.01071 31607920 PMC6761301

[186] Zeng AQ Yu Y Yao YQ Yang FF Liao M Song LJ. Betulinic acid impairs metastasis and reduces immunosuppressive cells in breast cancer models. Oncotarget. (2018) 9:3794–804. doi: 10.18632/oncotarget.23376 PMC5790500 29423083

[187] Vyas D Lopez-Hisijos N Shah P Deshpande KS Basson MD Vyas A. A second-generation proteasome inhibitor and doxorubicin modulates IL-6, pSTAT-3 and NF-kB activity in MDA-MB-231 breast cancer cells. J Nanosci Nanotechnol. (2017) 17:175–85. doi: 10.1166/jnn.2017.12427 29617099

[188] Cheng CC Shi LH Wang XJ Wang SX Wan XQ Liu SR. Stat3/Oct-4/c-Myc signal circuit for regulating stemness-mediated doxorubicin resistance of triple-negative breast cancer cells and inhibitory effects of WP1066. Int J Oncol. (2018) 53:339–48. doi: 10.3892/ijo.2018.4399 29750424

[189] El HH Saleh A Al SH Athamneh K Attoub S Arafat K. Rhus coriaria suppresses angiogenesis, metastasis and tumor growth of breast cancer through inhibition of STAT3, NFkappaB and nitric oxide pathways. Sci Rep. (2016) 6:21144. doi: 10.1038/srep21144 26888313 PMC4758048

[190] Li W Zhang H Nie M Tian Y Chen X Chen C. Ursolic acid derivative FZU-03,010 inhibits STAT3 and induces cell cycle arrest and apoptosis in renal and breast cancer cells. Acta Biochim Biophys Sin (Shanghai). (2017) 49:367–73. doi: 10.1093/abbs/gmx012 28338932

[191] Kim YJ Kim JY Lee N Oh E Sung D Cho TM. Disulfiram suppresses cancer stem-like properties and STAT3 signaling in triple-negative breast cancer cells. Biochem Biophys Res Commun. (2017) 486:1069–76. doi: 10.1016/j.bbrc.2017.03.164 28373070

[192] Dai X Yin C Guo G Zhang Y Zhao C Qian J. Schisandrin B exhibits potent anticancer activity in triple negative breast cancer by inhibiting STAT3. Toxicol Appl Pharmacol. (2018) 358:110–19. doi: 10.1016/j.taap.2018.09.005 30195018

[193] Dai X Yin C Zhang Y Guo G Zhao C Wang O. Osthole inhibits triple negative breast cancer cells by suppressing STAT3. J Exp Clin Cancer Res. (2018) 37:322. doi: 10.1186/s13046-018-0992-z 30577812 PMC6303899

[194] Qu Z Lin Y Mok DK Bian Q Tai WC Chen S. Brevilin A, a natural sesquiterpene lactone inhibited the growth of triple-negative breast cancer cells via akt/mTOR and STAT3 signaling pathways. Onco Targets Ther. (2020) 13:5363–73. doi: 10.2147/OTT.S256833 PMC7293987 32606754

[195] Qu Z Lin Y Mok DK Bian Q Tai WC Chen S. Arnicolide D inhibits triple negative breast cancer cell proliferation by suppression of akt/mTOR and STAT3 signaling pathways. Int J Med Sci. (2020) 17:1482–90. doi: 10.7150/ijms.46925 PMC7359397 32669950

[196] Zhu Z Yuan J Xu X Wei Y Yang B Zhao H. Eucannabinolide, a novel sesquiterpene lactone, suppresses the growth, metastasis and BCSCS-like traits of TNBC via inactivation of STAT3. Neoplasia. (2021) 23:36–48. doi: 10.1016/j.neo.2020.10.012 33217668 PMC7683293

[197] Byun WS Bae ES Cui J Park HJ Oh DC Lee SK. Antitumor activity of pulvomycin via targeting activated-STAT3 signaling in docetaxel-resistant triple-negative breast cancer cells. Biomedicines. (2021) 9:436. doi: 10.3390/biomedicines9040436 33920736 PMC8074004

[198] Zhu Y Yue P Dickinson CF Yang JK Datanagan K Zhai N. Natural product preferentially targets redox and metabolic adaptations and aberrantly active STAT3 to inhibit breast tumor growth in *vivo* . Cell Death Dis. (2022) 13:1022. doi: 10.1038/s41419-022-05477-2 36473850 PMC9726930

[199] An H Kim JY Oh E Lee N Cho Y Seo JH. Salinomycin promotes anoikis and decreases the CD44+/CD24- stem-like population via inhibition of STAT3 activation in MDA-MB-231 cells. PloS One. (2015) 10:e141919. doi: 10.1371/journal.pone.0141919 PMC4631341 26528725

[200] Bellat V Verchere A Ashe SA Law B. Transcriptomic insight into salinomycin mechanisms in breast cancer cell lines: synergistic effects with dasatinib and induction of estrogen receptor beta. BMC Cancer. (2020) 20:661. doi: 10.1186/s12885-020-07134-3 32678032 PMC7364656

[201] Mesmar J Abdallah R Hamade K Baydoun S Al-Thani N Shaito A. Ethanolic extract of Origanum Syriacum L. leaves exhibits potent anti-breast cancer potential and robust antioxidant properties. Front Pharmacol. (2022) 13:994025. doi: 10.3389/fphar.2022.994025 36299882 PMC9589507

[202] Lee HH Jung J Moon A Kang H Cho H. Antitumor and anti-invasive effect of apigenin on human breast carcinoma through suppression of IL-6 expression. Int J Mol Sci. (2019) 20:3143. doi: 10.3390/ijms20133143 31252615 PMC6651620

[203] Chuang HC Kapuriya N Kulp SK Chen CS Shapiro CL. Differential anti-proliferative activities of poly(ADP-ribose) polymerase (PARP) inhibitors in triple-negative breast cancer cells. Breast Cancer Res Treat. (2012) 134:649–59. doi: 10.1007/s10549-012-2106-5 PMC4297209 22678161

[204] Palandri F Tiribelli M Benevolo G Tieghi A Cavazzini F Breccia M. Efficacy and safety of ruxolitinib in intermediate-1 IPSS risk myelofibrosis patients: Results from an independent study. Hematol Oncol. (2018) 36:285–90. doi: 10.1002/hon.2429 28512865

[205] Al-Ali HK Griesshammer M Foltz L Palumbo GA Martino B Palandri F. Primary analysis of JUMP, a phase 3b, expanded-access study evaluating the safety and efficacy of ruxolitinib in patients with myelofibrosis, including those with low platelet counts. Br J Haematol. (2020) 189:888–903. doi: 10.1111/bjh.16462 32017044

[206] Gerds AT Tauchi T Ritchie E Deininger M Jamieson C Mesa R. Phase 1/2 trial of glasdegib in patients with primary or secondary myelofibrosis previously treated with ruxolitinib. Leuk Res. (2019) 79:38–44. doi: 10.1016/j.leukres.2019.02.012 30849661 PMC8148985

[207] Gerds AT Savona MR Scott BL Talpaz M Egyed M Harrison CN. Determining the recommended dose of pacritinib: results from the PAC203 dose-finding trial in advanced myelofibrosis. Blood Adv. (2020) 4:5825–35. doi: 10.1182/bloodadvances.2020003314 PMC7686901 33232476

[208] Mesa RA Vannucchi AM Mead A Egyed M Szoke A Suvorov A. Pacritinib versus best available therapy for the treatment of myelofibrosis irrespective of baseline cytopenias (PERSIST-1): an international, randomised, phase 3 trial. Lancet Haematol. (2017) 4:e225–36. doi: 10.1016/S2352-3026(17)30027-3 PMC8209752 28336242

[209] Verstovsek S Mesa R Talpaz M Kiladjian JJ Harrison CN Oh ST. Retrospective analysis of pacritinib in patients with myelofibrosis and severe thrombocytopenia. Haematologica. (2022) 107:1599–607. doi: 10.3324/haematol.2021.279415 PMC9244834 34551507

[210] Mascarenhas J Hoffman R Talpaz M Gerds AT Stein B Gupta V. Pacritinib vs best available therapy, including ruxolitinib, in patients with myelofibrosis: A randomized clinical trial. JAMA Oncol. (2018) 4:652–59. doi: 10.1001/jamaoncol.2017.5818 PMC5885169 29522138

[211] Foltz L Pica GM Zerazhi H Van Droogenbroeck J Visanica S Baez DLFE. Safety and efficacy findings from the open-label, multicenter, phase 3b, expanded treatment protocol study of ruxolitinib for treatment of patients with polycythemia vera who are resistant/intolerant to hydroxyurea and for whom no alternative treatments are available. Leuk Lymphoma. (2019) 60:3493–502. doi: 10.1080/10428194.2019.1636985 31359808

[212] Sylvine P Thomas S Pirayeh E. Infections associated with ruxolitinib: study in the French Pharmacovigilance database. Ann Hematol. (2018) 97:913–14. doi: 10.1007/s00277-018-3242-8 29340760

[213] Porpaczy E Tripolt S Hoelbl-Kovacic A Gisslinger B Bago-Horvath Z Casanova-Hevia E. Aggressive B-cell lymphomas in patients with myelofibrosis receiving JAK1/2 inhibitor therapy. Blood. (2018) 132:694–706. doi: 10.1182/blood-2017-10-810739 29907599 PMC7115916

[214] Wang P Zhou J. Proteolysis targeting chimera (PROTAC): A paradigm-shifting approach in small molecule drug discovery. Curr Top Med Chem. (2018) 18:1354–56. doi: 10.2174/1568026618666181010101922 30306871

[215] Noblejas-Lopez M Nieto-Jimenez C Burgos M Gomez-Juarez M Montero JC Esparis-Ogando A. Activity of BET-proteolysis targeting chimeric (PROTAC) compounds in triple negative breast cancer. J Exp Clin Cancer Res. (2019) 38:383. doi: 10.1186/s13046-019-1387-5 31470872 PMC6717344

[216] Li G Lin SS Yu ZL Wu XH Liu JW Tu GH. A PARP1 PROTAC as a novel strategy against PARP inhibitor resistance via promotion of ferroptosis in p53-positive breast cancer. Biochem Pharmacol. (2022) 206:115329. doi: 10.1016/j.bcp.2022.115329 36309080

[217] He R Song Z Bai Y He S Huang J Wang Y. Discovery of AXL degraders with improved potencies in triple-negative breast cancer (TNBC) cells. J Med Chem. (2023) 66:1873–91. doi: 10.1021/acs.jmedchem.2c01682 36695404

[218] Adams CM Mitra R Xiao Y Michener P Palazzo J Chao A. Targeted MDM2 degradation reveals a new vulnerability for p53-inactivated triple-negative breast cancer. Cancer Discovery. (2023) 13:1210–29. doi: 10.1158/2159-8290.CD-22-1131 PMC10164114 36734633

[219] Li X Zhang Z Gao F Ma Y Wei D Lu Z. c-myc-targeting PROTAC based on a TNA-DNA bivalent binder for combination therapy of triple-negative breast cancer. J Am Chem Soc. (2023) 145:9334–42. doi: 10.1021/jacs.3c02619 37068218

[220] Zhao C Chen D Suo F Setroikromo R Quax WJ Dekker FJ. Discovery of highly potent HDAC8 PROTACs with anti-tumor activity. Bioorg Chem. (2023) 136:106546. doi: 10.1016/j.bioorg.2023.106546 37098288

[221] Pu C Liu Y Deng R Xu Q Wang S Zhang H. Development of PROTAC degrader probe of CDK4/6 based on DCAF16. Bioorg Chem. (2023) 138:106637. doi: 10.1016/j.bioorg.2023.106637 37276679

[222] Yang J Chang Y Tien JC Wang Z Zhou Y Zhang P. Discovery of a highly potent and selective dual PROTAC degrader of CDK12 and CDK13. J Med Chem. (2022) 65:11066–83. doi: 10.1021/acs.jmedchem.2c00384 PMC9876424 35938508

[223] Niu T Li K Jiang L Zhou Z Hong J Chen X. Noncovalent CDK12/13 dual inhibitors-based PROTACs degrade CDK12-Cyclin K complex and induce synthetic lethality with PARP inhibitor. Eur J Med Chem. (2022) 228:114012. doi: 10.1016/j.ejmech.2021.114012 34864331

[224] Dale B Anderson C Park KS Kaniskan HU Ma A Shen Y. Targeting triple-negative breast cancer by a novel proteolysis targeting chimera degrader of enhancer of zeste homolog 2. ACS Pharmacol Transl Sci. (2022) 5:491–507. doi: 10.1021/acsptsci.2c00100 35837138 PMC9274772

[225] Wang C Chen X Liu X Lu D Li S Qu L. Discovery of precision targeting EZH2 degraders for triple-negative breast cancer. Eur J Med Chem. (2022) 238:114462. doi: 10.1016/j.ejmech.2022.114462 35623249

[226] Wu Y Xue J Li J. Chemical degrader enhances the treatment of androgen receptor-positive triple-negative breast cancer. Arch Biochem Biophys. (2022) 721:109194. doi: 10.1016/j.abb.2022.109194 35337811

[227] Pu C Tong Y Liu Y Lan S Wang S Yan G. Selective degradation of PARP2 by PROTACs via recruiting DCAF16 for triple-negative breast cancer. Eur J Med Chem. (2022) 236:114321. doi: 10.1016/j.ejmech.2022.114321 35430559

[228] Watson IR Irwin MS Ohh M. NEDD8 pathways in cancer, Sine Quibus Non. Cancer Cell. (2011) 19:168–76. doi: 10.1016/j.ccr.2011.01.002 21316600

